# Ciliary Type III Adenylyl Cyclase in the VMH Is Crucial for High‐Fat Diet‐Induced Obesity Mediated by Autophagy

**DOI:** 10.1002/advs.202102568

**Published:** 2021-11-16

**Authors:** Dong Yang, Xiangbo Wu, Weina Wang, Yanfen Zhou, Zhenshan Wang

**Affiliations:** ^1^ College of Life Science Institute of Life Science and Green Development Hebei University Baoding Hebei 071002 China

**Keywords:** autophagy, cilia, obesity, type III adenylyl cyclase, ventromedial hypothalamus

## Abstract

Neuronal primary cilia are crucial for body weight maintenance. Type III adenylyl cyclase (AC3) is abundantly enriched in neuronal cilia, and mice with global AC3 ablation are obese. However, whether AC3 regulates body weight through its ciliary expression and the mechanism underlying this potential regulation are not clear. In this study, humanized AC3 knock‐in mice that are resistant to high‐fat diet (HFD)‐induced obesity are generated, and increases in the number and length of cilia in the ventromedial hypothalamus (VMH) are shown. It is demonstrated that mice with specifically knocked down ciliary AC3 expression in the VMH show pronounced HFD‐induced obesity. In addition, in vitro and in vivo analyses of the VMH show that ciliary AC3 regulates autophagy by binding an autophagy‐related gene, gamma‐aminobutyric acid A receptor‐associated protein (GABARAP). Mice with GABARAP knockdown in the VMH exhibit exacerbated HFD‐induced obesity. Overall, the findings may reveal a potential mechanism by which ciliary AC3 expression regulates body weight in the mouse VMH.

## Graphical abstract

Type III adenylyl cyclase (AC3) is abundantly enriched in neuronal cilia. This paper reports that ciliary AC3 in the ventromedial hypothalamus (VMH) is essential to high‐fat diet (HFD)‐induced obesity. Cell lines and mouse models show that in the VMH, ciliary AC3 regulates autophagy by binding an autophagy‐related gene, gamma‐aminobutyric acid A receptor‐associated protein (GABARAP). Mice with GABARAP knockdown in the VMH exhibit exacerbated HFD‐induced obesity.

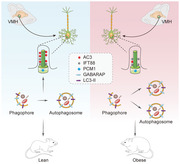

## 1 Introduction

Almost all nondividing neurons of the central nervous system are equipped with a primary cilium that acts as a sensory antenna projected from the cell surface. It senses signaling molecules in the extracellular environment and synergistically relays messages to the cell interior to coordinate cytoplasmic effectors.^[^ ^1^, ^2^ ^]^ Defects in cilia contribute to a group of disorders known collectively as ciliopathies, which include obesity.^[^ ^3^, ^4^ ^]^ However, the molecular mechanisms underlying obesity resulting from a dysfunction of ciliary structure or signaling remain unknown.

G protein‐coupled receptors (GPCRs) are highly versatile membrane sensors that respond to diverse extracellular signals and transmit them to the cell interior via G‐proteins and downstream effectors.^[^ ^5^ ^]^ GPCR‐stimulated 3′,5′‐cyclic adenosine monophosphate (cAMP) production requires three essential components of the signaling cascade: GPCRs, G proteins, and adenylyl cyclases (ACs). As a downstream component of GPCR signaling cascades, abundant type III adenylyl cyclase (AC3) is concentrated in brain neurons and is commonly employed as a universal neuronal ciliary marker.^[^ ^6^ ^]^ In addition, GPCRs and AC3 are colocalized in neuronal cilia to mediate cAMP signaling in response to GPCR activation. In a previous study, we revealed that mice with global AC3 knockout (AC3^−/−^) showed hyperphagia‐induced obesity.^[^ ^7^ ^]^ Conversely, an AC3 gain‐of‐function mutation protects mice from diet‐induced obesity.^[^ ^8^ ^]^ These results strongly suggest that AC3 is crucial for body weight maintenance. However, why AC3^−/−^ mice are obese remains unclear.

The ventromedial hypothalamus (VMH) within the hypothalamus is a satiety‐sensing center responsible for regulating food intake and body weight.^[^ ^9^, ^10^, ^11^ ^]^ Hypothalamic neuronal cilia are essential for sensing and regulating the systemic energy status. Although AC3 is implicated in the propagation of obesity and is abundantly expressed in the neuronal cilia of the VMH,^[^ ^7^ ^]^ direct experimental evidence in support of a link between these two events has yet to be reported.

Autophagy is a cellular process through which cells engulf and degrade damaged cytoplasmic components. Autophagy is very sensitive to changes in cellular nutritional status, especially the intake of high‐fat, high‐calorie food.^[^ ^12^ ^]^ In particular, studies in animals have demonstrated the involvement of hypothalamic autophagy in the regulation of food intake and energy expenditure.^[^ ^13^, ^14^, ^15^, ^16^, ^17^, ^18^ ^]^ Paralleling the dynamic exchange of signaling molecules between the extracellular environment and the cell interior, both ciliary and autophagic processes must be appropriately regulated depending on the model of change. Thus, it is not surprising that the cilia and autophagy can engage in crosstalk and reciprocally interact with each other.

The aim of this study was to utilize a set of novel mouse models that allowed VMH cilia‐specific AC3 knockdown (KD) or overexpression of human AC3 in mice to investigate whether ciliary AC3 expression in the VMH functioned as a key driver of the exaggerated development of obesity under conditions of HFD consumption. In addition, the contribution of weight regulated by ciliary AC3 expression within the VMH to the autophagy process was also investigated.

## 2 Results

### 2.1 hAC3 Knock‐In Mice Are Resistant to High‐Fat Diet‐Induced Obesity and Exhibit an Increase in the Number and Length of Cilia in the VMH

Genome‐wide association studies (GWAS) have revealed that human AC3 (hAC3) is associated with obesity.^[^ ^19^, ^20^, ^21^, ^22^, ^23^, ^24^, ^25^, ^26^, ^27^ ^]^ To examine the function of human AC3 (hAC3) in regulating body weight, we generated a humanized mouse by introducing the complete hAC3 sequence into the Rosa26 site in the mouse genome (Figure S1, Supporting Information). The resulting humanized AC3 knock‐in mice (hAC3 mice) were fertile and healthy throughout their lifespan.

**Figure 1:**
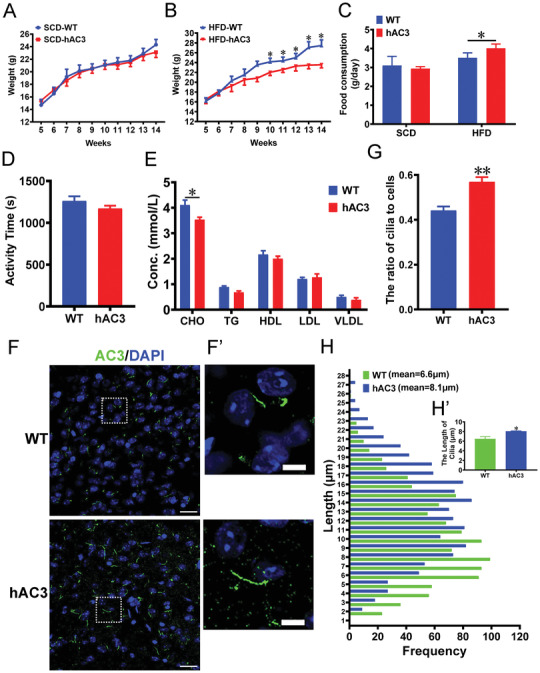
hAC3 mice with resistance to HFD‐induced obesity exhibit increased numbers and lengths of cilia in the VMH. A,B) The growth curves of SCD‐fed WT, SCD‐fed hAC3, HFD‐fed WT, and HFD‐fed hAC3 mice starting at 5 weeks of age (*n* = 10 mice per group). C) Twenty‐four hours of food consumption in WT and hAC3 mice (*n* = 10 mice per group). D) Total physical activity of WT and hAC3 mice (*n* = 10 mice per group). E) Hormone levels in the serum of WT and hAC3 mice (*n* = 6 mice per group). F) Representative images showing AC3^+^ cilia in the VMHs of WT and hAC3 mice. F′) A higher magnification of the boxed region. Scale bars: F) 20 µm; F′) 5 µm. G) Quantification of the number of cilia in the VMHs of WT and hAC3 mice in HFD feeding condition (*n* = 4 mice per group). H) Quantification of the length of cilia in the VMHs of WT and hAC3 mice in HFD feeding condition (*n* = 4 mice per group). Data represent the means ± SEM; ^***^ *p* < 0.0001; ^*^ *p* < 0.05; Student's *t*‐test or one‐way ANOVA and Bonferroni pairwise comparisons.

The body weights of the hAC3 mice and their wild‐type (WT) littermates were measured weekly beginning at the age of 3 weeks. The two mouse lines did not show a significant difference in body weight under standard chow diet (SCD) feeding (**Figure** **1**A). We then fed the mice a HFD (45% fat) and assessed their weight weekly from the 3rd to 14th week after birth. The weight curves of the hAC3 and WT mice showed that the group weights diverged after seven weeks of HFD feeding (Figure 1B). After 12 weeks of HFD intake, the weight of the hAC3 mice had increased by 46.12%, whereas that of the WT mice had increased by 65.32% (Figure 1B), suggesting that hAC3 mice show a slower transition to obesity than WT mice. The SCD intake was not significantly different between the groups; however, HFD intake was significantly higher in hAC3 mice than in the controls at the 14th week after birth (Figure 1C). Moreover, physical activity was not significantly different between hAC3 and WT mice (Figure 1D). The total triglyceride (TG) content in plasma was significantly reduced, whereas cholesterol (CHO) and lipoprotein levels were not different between hAC3 mice and WT mice (Figure 1E).

AC3 is highly expressed in the cilia of the VMH, which is enriched in longer cilia relative to other brain areas.^[^ ^28^, ^29^ ^]^ Therefore, we explored the number and length of the cilia in the VMH in hAC3 mice. Immunofluorescence (IF) showed that the percentage of AC3^+^ cilia was significantly higher and that the cilia were longer in the VMHs of hAC3 mice than in those of WT mice (Figure 1F–H). Taken together, our results confirm that hAC3 mice are resistant to HFD‐induced obesity, which is accompanied by increases in the length and number of cilia in the VMH.

### 2.2 Mice with AC3 KD in the VMH Are More Susceptible to HFD‐Induced Obesity

**Figure 2:**
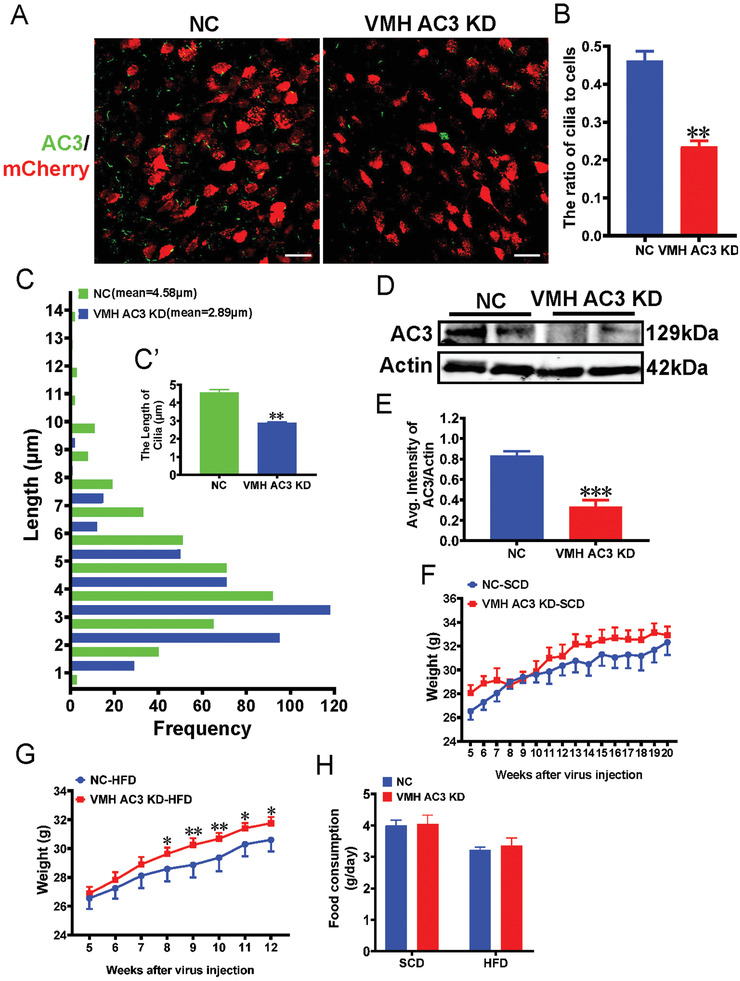
VMH AC3 KD mice exhibit increased susceptibility to HFD‐induced obesity. A) Representative images showing the expression of AC3 in the VMHs of VMH AC3 KD mice and the controls. Scale bar: 20 µm. B) Quantification of the number of cilia in the VMHs of VMH AC3 KD mice and controls (*n* = 3 mice per group). C) Quantification of the length of cilia in the VMHs of VMH AC3 KD mice and controls (*n* = 3 mice per group). D, E) Western blot (WB) (D) and densitometric quantification (E) of AC3 expression in the hypothalami of VMH AC3 KD mice and the controls. Actin served as a loading control (*n* = 3 mice per group). F, G) The growth curves of NC and VMH AC3 KD mice starting at 5 weeks after virus injection under SCD feeding (F) and HFD feeding (G) conditions (*n* = 12 mice per group). H) Twenty‐four hours of food consumption in the VMH AC3 KD mice and controls (*n* = 12 mice per group). Data represent the mean ± SEM; ^*^ *p* < 0.05, ^**^ *p* < 0.01, and ^***^ *p* < 0.001; Student's *t*‐test or one‐way ANOVA and Bonferroni pairwise comparisons.

To better understand the role of AC3 in the VMH, we knocked down AC3 specifically in the VMH via the stereotactic delivery of an adeno‐associated virus (AAV) mixture (1:1 ratio) of pMecp2‐spCas9 and AC3 sgRNA‐mCherry into the VMH in 4‐week‐old male WT mice [VMH AC3 KD], while mice injected with pMecp2‐spCas9+normal control (NC) sgRNA‐mCherry AAV served as controls (VMH NC mice) (Figure S2A, Supporting Information). We observed an ≈90% AAV infection efficiency in the VMH at 6 weeks after virus injection (Figure S2A, Supporting Information). The number of AC3^+^ cilia in the VMH was decreased by ≈50%, and the cilia were shorter in the VMHs of VMH AC3 KD mice than in those of NC mice (**Figure** **2**A–C). In addition, the total AC3 protein content was decreased by over 60% in the VMH AC3 KD mice relative to the corresponding controls (Figure 2D,E). However, in areas surrounding the VMH, including the paraventricular nucleus (PVN), dorsal medial hypothalamic nucleus (DMH), and arcuate nucleus (ARC), the levels of AC3 were indistinguishable between VMH AC3 KD mice and the controls (Figure S2B, Supporting Information). In addition, off‐target editing was not observed in the VMH AC3 KD mice (Figure S2C, Supporting Information)

Body weight observations showed that under SCD feeding conditions, there was no difference between VMH AC3 KD mice and the controls over a period of twenty weeks after AAV injection (Figure 2F). We exposed VMH AC3 KD mice to an HFD after AAV injection, and their weight was assessed weekly over a period of 12 weeks. The weight curves of the VMH AC3 KD mice and NC mice showed that their weights diverged 8 weeks after virus injection (Figure 2G). By the 12th week after virus injection, the weight of the HFD‐fed NC mice had increased by 47.54%. However, the weight of the HFD‐fed VMH AC3 KD mice had increased by 58.80% (Figure 2G). No differences in food intake were observed between the groups under SCD and HFD feeding conditions in the 12th week after virus injection (Figure 2H). These results showed that VMH AC3 KD mice are more susceptible to HFD‐induced obesity than control mice.

### 2.3 Ciliary AC3 in the VMH Is Essential for Body Weight Maintenance under HFD Feeding Conditions

**Figure 3:**
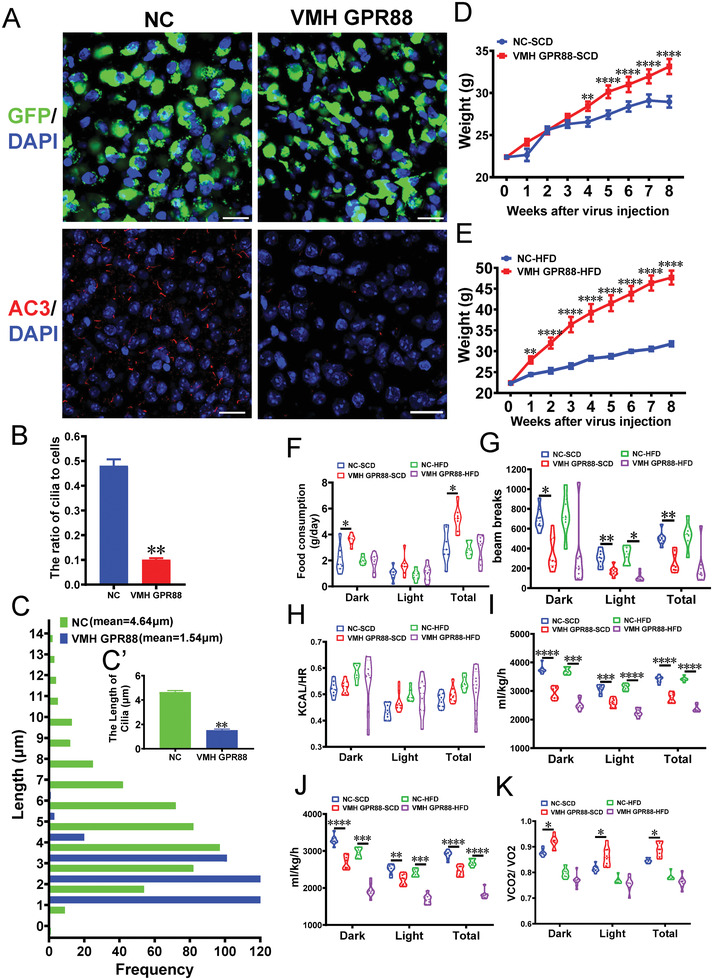
VMH AAV GPR88* mice show obesity symptoms under SCD and HFD feeding conditions. A) Representative images showing GFP^+^ and AC3^+^ cilia in the VMHs of VMH AAV GPR88* mice and the controls. Scale bar: 20 µm. B) Quantification of the number of cilia in the VMHs of VMH GPR88* mice and controls (*n* = 3 mice per group). C) Quantification of the length of cilia in the VMHs of VMH GPR88* mice and controls (*n* = 3 mice per group). D, E) The growth curves of NC and VMH AAV GPR88* mice starting after virus injection under SCD feeding (D) and HFD feeding (E) conditions (*n* = 8 mice per group). F) Twenty‐four hours of food consumption by NC and VMH AAV GPR88* mice (*n* = 8 mice per group). G‐K) Total activity (XTOT, G), HEAT (H), VO2 (I), VCO2 (J), and RER (RER = VCO2/VO2, K) of VMH AAV GPR88* mice and the controls (*n* = 8 mice per group).

We subsequently specifically knocked down AC3 expression in the cilia of the VMH. Specific inhibition of AC3 in the cilia of neurons can presumably be achieved by the expression of a constitutively active version of the specific G*α*i/o‐protein‐coupled receptor GPR88 (GPR88 p. Gly283His, denoted GPR88*).^[^ ^30^ ^]^ Thus, we fused mutated GPR88 (GPR88*) coding sequences (CDSs) with pSyn2‐GFP and then packaged these constructs into AAV. AAV carrying only pSyn2‐GFP was treated as a control. Mice aged 4 weeks were stereotactically injected with pSyn2‐GPR88*‐GFP AAV or pSyn2‐GFP AAV in the VMH (Figure S3A, Supporting Information). Six weeks after AAV injection, we observed an ≈90% AAV infection efficiency in the VMH in mice (**Figure** **3**A; Figure S3B, Supporting Information). IF results showed that the number of AC3^+^ cilia was significantly reduced in the VMH but was not altered in the PVN, DMH, or ARC of AAV GPR88* mice relative to the controls (Figure 3A,B; Figure S3C, Supporting Information). In addition, the cilia were shorter in the VMHs of VMH AAV GPR88* mice than in those of NC mice (Figure 3A,C).

Under SCD feeding conditions, the weight curves diverged between VMH AAV GPR88* mice and the controls four weeks after AAV injection (Figure 3D; Figure S3D, Supporting Information). By eight weeks after AAV injection, the weight gain of the NC mice had reached 29.25%, whereas that of the VMH AAV GPR88* mice had reached 47.95% (Figure 3D). Under HFD feeding conditions, the weight curves diverged between the VMH AAV GPR88* mice and the controls only one week after AAV injection (Figure 3E; Figure S3D, Supporting Information). After eight weeks of HFD intake, the weight of the NC mice had increased by 42.12%, whereas that of the VMH AAV GPR88* mice had increased by 112.77% (Figure 3E). Physical activity was lower and SCD intake was higher in the VMH AAV GPR88* mice than in the controls, although their HFD intake did not differ (Figure 3F,G). Although thermogenesis showed no change, oxygen consumption and carbon dioxide production were lower in the VMH AAV GPR88* mice (Figure 3H–J). The respiratory exchange ratio (RER; VCO2/VO2) was higher in the VMH AAV GPR88* mice under SCD feeding conditions but did not differ between the groups under HFD feeding conditions (Figure 3K). Fasting plasma levels of CHO, high‐density lipoprotein (HDL), and low‐density lipoprotein (LDL) were significantly higher in the VMH AAV GPR88* mice than in the controls (Figure S3E, Supporting Information). Taken together, these results indicated that VMH AAV GPR88* mice are subject to obesity accompanied by lower energy expenditure.

**Figure 4:**
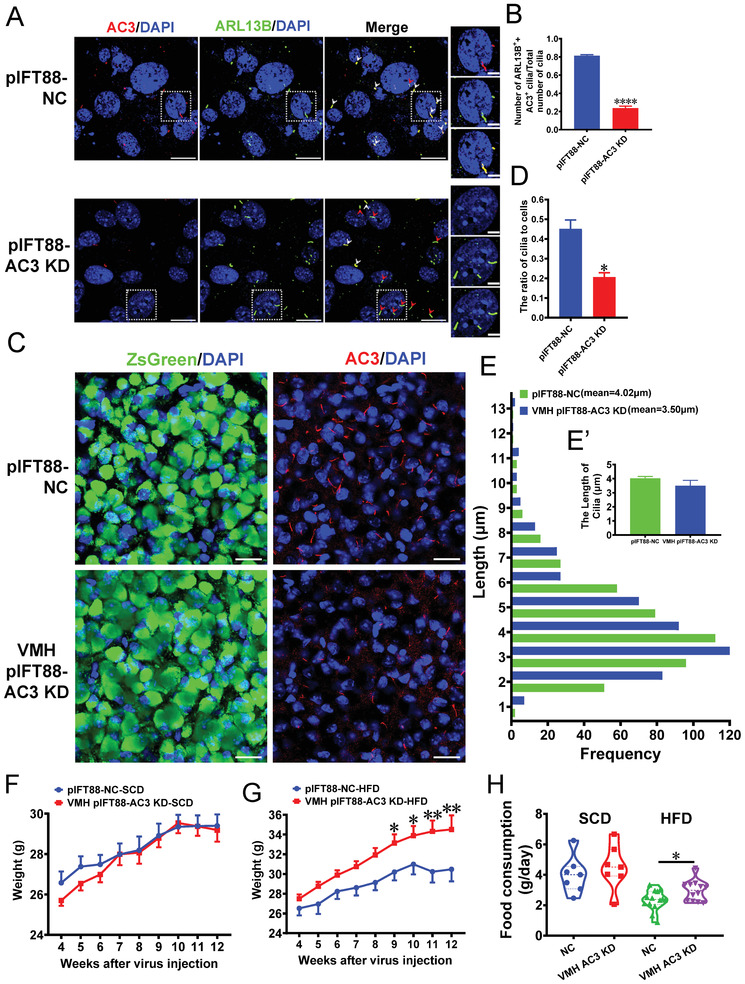
VMH pIFT88‐AC3 KD mice show increasing weight under HFD feeding conditions. A) Representative IF co‐staining with AC3 and ARL13B antibodies in MEFs transfected with LV‐pIFT88‐shAC3 and the controls. Scale bars: 20 and 5 µm. B) Quantification of the number of ARL13B‐ and AC3‐positive cilia/the number of total cilia in MEFs transfected with LV‐pIFT88‐shAC3 and the controls (*n* = 3 repetitions per group). C) Representative images showing AC3^+^ cilia in the VMHs of VMH pIFT88‐AC3 KD mice and the controls. Scale bars: 20 µm. D) Quantification of the number of cilia in the VMHs of VMH pIFT88‐AC3 KD mice and controls (*n* = 3 mice per group). E) Quantification of the length of cilia in the VMHs of VMH pIFT88‐AC3 KD mice and controls (*n* = 3 mice per group). F, G) The growth curve of NC and pIFT88‐AC3 KD mice starting 4 weeks after virus injection under SCD feeding (F) and HFD feeding (G) conditions (*n* = 10 mice per group). H) Twenty‐four hours of food consumption in NC and pIFT88‐AC3 KD mice (SCD: *n* = 7 mice, HFD: *n* = 14 mice). Data represent the mean ± SEM; ^*^ *p* < 0.05, ^**^ *p* < 0.01, and ^****^ *p* < 0.0001; Student's *t*‐test or one‐way ANOVA and Bonferroni pairwise comparisons.

GPR88 is mainly expressed on the cytoplasmic membrane of neurons and colocalizes with NeuN,^[^ ^31^ ^]^ and AC3 is mainly expressed on the cilia of neurons.^[^ ^6^ ^]^ GPR88* inhibits cAMP accumulation in both the cytoplasmic and ciliary compartments,^[^ ^32^ ^]^ implying that the phenotypes of the VMH AAV GPR88* mice might be caused by genes other than AC3 (see Section 3). This possibility prompted us to identify more specific tools for knocking down ciliary AC3 in the VMH. Intraflagellar transport 88 (IFT88) is considered to be specifically expressed on the axonemes of cilia.^[^ ^33^ ^]^ We assessed whether a short cilium‐specific IFT88 promoter could achieve efficient ciliary AC3 KD. The results of experiments in 293T and Murine Embryonic Fibroblast (MEF) cells showed that the IFT88 promoter could be used to drive the expression of genes (Figure S4, Supporting Information) and that the pIFT88‐AC3 shRNA construct could specifically knock down ciliary AC3 expression (**Figure** **4**A,B; Figure S5A,B Supporting Information). Since the combined size of the IFT88 promoter and spCas9 is far greater than the maximum AAV carrying capacity,^[^ ^34^ ^]^ we chose pIFT88‐AC3 shRNA for AAV packaging to achieve specific AC3 KD in the cilia of the VMH.

Next, we injected AAV containing pIFT88‐shAC3‐ZsGreen into the VMHs of 4‐week‐old mice to specifically knock down AC3 expression in cilia (VMH pIFT88‐AC3 KD mice), and AAV‐pIFT88‐shNC‐ZsGreen‐injected mice were used as controls. Six weeks after AAV injection, the AAV infection efficiency was >90% (Figure 4C). IF staining showed that the number of AC3^+^ cilia was significantly reduced in the VMH of pIFT88‐AC3 KD mice (Figure 4C,D), whereas it was not altered in the PVN, DMH, or ARC (Figure S6, Supporting Information). The length of the cilia was not significantly different in the VMHs between the pIFT88‐AC3 KD and pIFT88‐NC mice (Figure 4C,E), indicating that AC3 was specifically knocked down in the cilia of the VMH.

Mice were weighed weekly after AAV stereotaxic injection. The SCD‐fed VMH pIFT88‐AC3 KD mice did not display significantly increased weight relative to the controls over a period of twelve weeks after AAV injection (Figure 4F), and food intake did not differ between the groups (Figure 4H). We subsequently examined whether the mice exhibited obesity induced by HFD feeding. Eight weeks after AAV injection, the weight curves diverged between the VMH pIFT88‐AC3 KD mice and the controls (Figure 4G ). By the 12th week, the weight of the HFD‐fed NC mice had increased by 40.42%; however, the weight of the HFD‐fed VMH pIFT88‐AC3 KD mice had increased by 62.92% over that at the time of AAV injection (Figure 4G ). In addition, the weight of the VMH pIFT88‐AC3 KD mice at this time point was 15.03% higher than that of the control group (Figure 4G ), which was accompanied by a significantly higher HFD intake (Figure 4H). Considering the results together, we concluded that the VMH pIFT88‐AC3 KD mice exhibited more severe HFD‐induced obesity than the controls, although their obesity phenotypes were not as pronounced as those of the VMH AAV GPR88* mice.

### 2.4 The Regulation of Autophagy by AC3 Is Cilium Dependent

**Figure 5:**
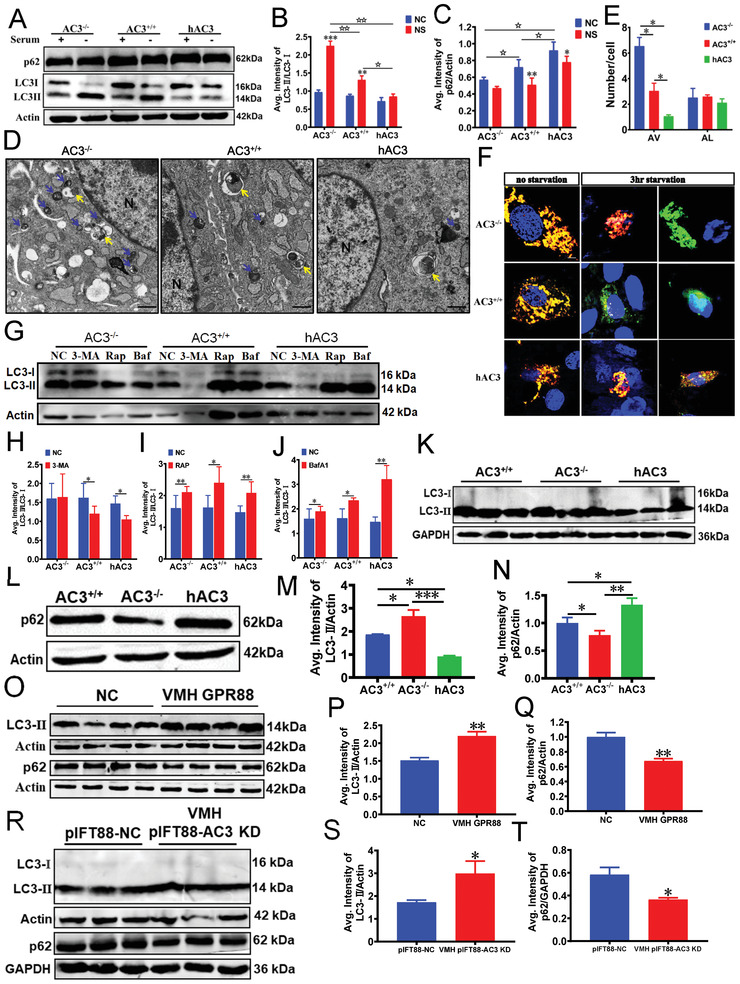
Autophagy is regulated by ciliary AC3 in MEFs and the VMH. A‐C) WB (A) and densitometric quantification of the expression of LC3 (B) and p62 (C) in AC3^+/+^, AC3^−/−^, and hAC3 MEFs with or without serum addition (*n* = 3 repetitions per group). Actin served as the loading control. D) Ultrastructural images of autophagic vacuoles (AV) and autolysosomes (AL) in AC3^+/+^, AC3^−/−^, and hAC3 MEFs were obtained by TEM. The blue arrow indicates AV. The yellow arrow indicates AL. N represents the nucleus. Scale bars: 2 µm. E) The numbers of autophagic vacuoles (AV) and autolysosomes (AL) in AC3^+/+^, AC3^−/−^, and hAC3 MEFs (*n* = 3 repetitions per group). F) Live‐cell imaging of AC3^+/+^, AC3^−/−^, and hAC3 MEFs transduced with the tandem mRFP‐GFP‐LC3 plasmid under treatment without serum or with 100 × 10^−9^ m bafilomycin A_1_ treatment. Blue, nuclei; green, GFP; red, REP. Scale bars: 5 µm. G‐J) WB (G) and densitometric quantification of the expression of LC3 in AC3^+/+^, AC3^−/−^, and hAC3 MEFs treated with 3‐MA (H), rapamycin (I), and bafilomycin A_1_ (J, *n* = 3 repetitions per group). GAPDH served as loading controls. K‐N) WB and densitometric quantification of the expression of LC3 (K, M) and p62 (L, N) in the hypothalami of AC3^+/+^, AC3^−/−^, and hAC3 mice (*n* = 3 repetitions per group). Actin and GAPDH served as loading controls. O‐Q) WB (O) and densitometric quantification of the expression of LC3 (P) and p62 (Q) in the hypothalami of VMH AAV GPR88* mice and the controls (*n* = 4 mice per group). Actin served as the loading control. R‐T) WB (R) and densitometric quantification of the expression of LC3 (S) and p62 (T) in the hypothalami of VMH pIFT88‐AC3 KD mice and the controls (*n* = 3 mice per group). GAPDH and actin served as loading controls. Data represent the mean ± SEM; ^*^ *p* < 0.05, ^**^ *p* < 0.01, and ^***^ *p* < 0.001; ^☆^ *p* < 0.05 and ^☆☆^ *p* < 0.01; Student's *t*‐test or one‐way ANOVA and Bonferroni pairwise comparisons.

Cilia and autophagy are regulated reciprocally, which is associated with the cAMP pathway.^[^ ^35^, ^36^, ^37^, ^38^, ^39^ ^]^ Hence, we speculated that ciliary AC3 might also regulate autophagy in mice. We first used AC3^−/−^, AC3^+/+^, and hAC3 MEFs to determine the role of AC3 in autophagy. WB analysis, ultrastructural analyses with transmission electron microscopy and mRFP–GFP–LC3 reporter analysis confirmed that AC3 regulates autophagic activity in MEFs (**Figure** **5**A–F). Subsequently, we used 3‐MA (an inhibitor of the early stage of autophagy), rapamycin (a potent and specific mTOR inhibitor), and bafilomycin A1 (BafA1, a selective inhibitor of autophagosome and lysosome fusion) to treat AC3^−/−^, AC3^+/+^, and hAC3 MEFs. WB results indicated that AC3 regulated autophagy in the stage of autophagosome formation (Figure 5G–J). Moreover, WB analysis showed that reduced AC3 expression in MEFs under serum‐free conditions led to increased autophagy (Figure S6A–C, Supporting Information).

Next, we used WB analysis to determine the levels of LC3 and p62 in hypothalami dissected from adult AC3^+/+^, AC3^−/−^, and hAC3 mice. The results revealed that there was a significant increase in LC3‐II and a decrease in p62 in the hypothalami of AC3^−/−^ mice compared with those of AC3^+/+^ mice (Figure 5K–N). Conversely, a decrease in LC3‐II and an increase in p62 were observed in the hypothalami of hAC3 mice compared to those of AC3^+/+^ mice (Figure 5K–N). In addition, IF analysis revealed a significant increase in the LC3‐II positive signal in VMH of AC3^−/−^ mice and a significant decrease in the VMH in hAC3 mice, as compared with AC3^+/+^ mice (Figure S7A,B, Supporting Information). An increase in the LC3‐II positive signal was also observed in the VMH of VMH AC3 KD mice compared with their controls (Figure S7C,D, Supporting Information). These results imply that autophagic activity is negatively regulated by AC3 in the hypothalamus.

To further explore whether the regulation of autophagic activity by AC3 is cilium dependent, we examined the levels of LC3 and p62 in the VMHs of AAV GPR88* mice and pIFT88‐AC3 KD mice. Western blot analyses showed that the LC3‐II level was significantly higher, whereas the p62 level was significantly lower in the hypothalami of AAV GPR88* mice than in those of the controls (Figure 5O–Q). Similarly, WB of the hypothalami of pIFT88‐AC3 KD mice showed that the LC3‐II level was significantly increased while the p62 level was significantly decreased relative to the controls (Figure 5R–T). Furthermore, IF analysis revealed a significant increases in the LC3‐II positive signal in the VMH of AAV GPR88* and pIFT88‐AC3 KD mice compared with their controls (Figure S8D–G, Supporting Information).

Taken together, the MEF and hypothalamus results revealed that autophagic activities are regulated by AC3 in a cilium‐dependent manner.

### 2.5 The Autophagy‐Related Gene Gamma‐Aminobutyric Acid A Receptor‐Associated Protein (GABARAP) Interacts with AC3 via LC3‐Interacting Regions (LIRs) in a Ciliary Expression‐Dependent Manner

**Figure 6:**
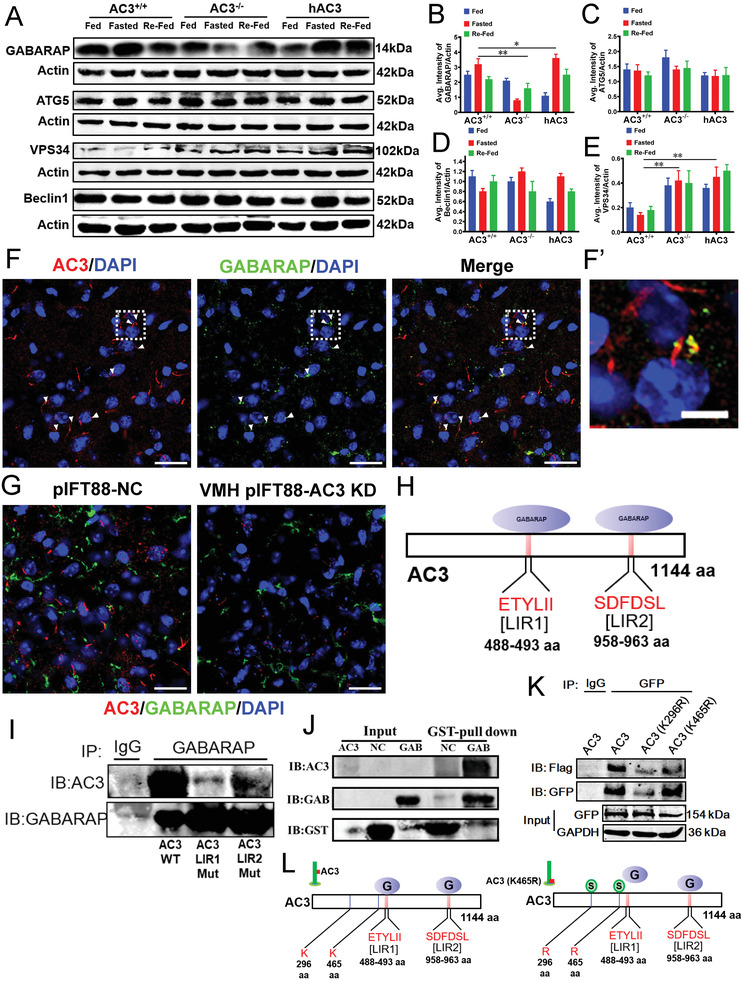
GABARAP interacts with AC3 via LIRs of AC3 in a ciliary expression‐dependent manner. A‐E) WB (A) and densitometric quantification of the expression of GABARAP (B), ATG5 (C), VPS34 (D), and Beclin1(E) in the hypothalami of AC3^+/+^, AC3^−/−^, and hAC3 mice (*n* = 3 mice per group). Actin served as the loading control. F) Representative IF co‐staining with AC3 and GABARAP antibodies in the VMHs of WT mice. F′) A higher magnification of the boxed region. Scale bars: F) 20 µm; F′) 5 µm. G) Representative images showing the expression levels of GABARAP and AC3 in the VMHs of VMH pIFT88‐AC3 KD mice and the controls. Scale bars: 20 µm. H) Schematic representation of GABARAP binding LIRs at aa488‐aa493 and aa958‐aa963 of AC3. I) Co‐IP analysis of GABARAP and AC3 (WT), AC3 (LIR1 Mut), or AC3 (LIR2 Mut). LgG served as the negative control. J) Pull‐down analysis of GABARAP and AC3. K) Co‐IP analysis of GABARAP and AC3 (WT), AC3 (296 Mut), or AC3 (465 Mut). LgG served as the negative control. L) Schematic representation of AC3 regulating GABARAP. Data represent the mean ± SEM; ^*^ *p* < 0.05 and ^**^ *p* < 0.01; one‐way ANOVA and Bonferroni pairwise comparisons.

Although our results showed that the alteration of AC3 ciliary expression affected autophagy activity, the underlying mechanism remains unknown. Autophagy‐related genes (ATGs) are regulated by cAMP,^[^ ^37^, ^40^ ^]^ and several ATGs (GABARAP, LC3, AMBRA1, VPS15, ATG16L, ATG5, VPS34, Atg7, ATG14, and Beclin1) exhibit ciliary expression.^[^ ^41^ ^]^ Therefore, to explore the mechanisms by which ciliary AC3 affected autophagy, we first used qPCR to determine the expression difference in ciliary‐associated ATGs in the hypothalamus of AC3^−/−^, AC3^+/+^, and hAC3 mice. The data revealed that both GABARAP and Beclin1 were significantly reduced, whereas ATG5 and VPS34 were dramatically increased, in the hypothalamus of AC3^−/−^ mice relative to the AC3^+/+^ controls (Figure S9, Supporting Information). Conversely, both GABARAP and Beclin1 were significantly increased, and ATG5 and VPS34 were dramatically reduced, in the hypothalamus of hAC3 knock‐in mice compared with the AC3^+/+^ animals (Figure S9, Supporting Information). The expression of other associated ATGs in the hypothalamus was indistinguishable among these animal groups (Figure S9, Supporting Information). Next, we extracted proteins from the hypothalamus of AC3^+/+^, AC3^−/−^, and hAC3 knock‐in mice for WB analyses. The results showed that GABARAP was significantly reduced and VPS34 was dramatically increased (**Figure** **6**A–E). The levels of both ATG5 and Beclin1 were not altered in the hypothalamus of AC3^−/−^ mice relative to AC3^+/+^ mice under fasting conditions (Figure 6A–E). The levels of GABARAP, VPS4, and Beclin1 were significantly increased, whereas ATG5 was not altered, in the hypothalamus of hAC3 knock‐in mice under fasting conditions compared with AC3^+/+^ mice (Figure 6A–E). Taken together, these results suggested that only GABARAP was positively regulated by AC3 at both mRNA and protein levels in the hypothalamus.

We then analyzed whether the interaction between AC3 and GABARAP is cilium dependent. IF staining showed that GABARAP was indeed expressed in the cilia of the VMH and partially colocalized with AC3 in cilia (Figure 6F). Subsequently, we examined the expression status of GABARAP in the VMH of pIFT88‐AC3 KD mice. IF staining showed that the GABARAP level was reduced in the VMH of pIFT88‐AC3 KD mice compared with their controls (Figure 6G), indicating that ciliary AC3 expression is required for GABARAP stabilization in the VMH.

The LC3/GABARAP family plays a pivotal role in autophagic activity by interacting with adaptor proteins, mainly via LIRs or GABARAP interaction motifs (GIMs).^[^ ^42^ ^]^ The LIR/GIM ensures that protein complexes are recruited to autophagosomes to perform their functions.^[^ ^43^, ^44^ ^]^ By searching the iLIR database (https://ilir.warwick.ac.uk/), we identified two LIR motifs (aa 488‐ETYLII aa 493 and 958 aa‐SDFDSL aa 963) in the AC3 amino acid (AA) sequence (Figure 6H), implying that autophagic regulation by ciliary AC3 may be mediated by binding to GABARAP. To test this possibility, we generated mutant constructs of AC3 in which the potential LIR/GIM sequences were replaced with alanine. Then, coimmunoprecipitation (co‐IP) was performed in NIH3T3 cells. The results showed that AC3 interacts with GABARAP, as illustrated by a remarkable reduction in the interaction of LIR/GIM mutant AC3 with GABARAP (Figure 6I). GST pull‐down assays further confirmed the interactive relationship between AC3 and GABARAP (Figure 6J). Clearly, the LIR/GIM sequences of AC3 are essential for the interaction with GABARAP.

To determine whether the interaction of AC3 with GABARAP is influenced by its ciliary expression, we mutated AC3 AA 296 or 465, as these two sumoylation sites normally undergo SUMO modification to allow AC3 to be transported to the ciliary axoneme. When these sites are mutated, AC3 cannot be sumoylated and therefore cannot be transported to the ciliary axoneme.^[^ ^51^ ^]^ Thus, the mutant AC3 cDNAs (AC3K296R or AC3K465R) were individually cotransfected with GABARAP cDNA into NIH3T3 cells, and co‐IP assays showed that binding to GABARAP was remarkably diminished with the mutation of the AA465 site in AC3 (Figure 6K), whereas the binding ability of AC3K296R with GABARAP was not altered relative to that of WT AC3 (Figure 6K). Given the accumulating evidence indicating that GABARAP is essential for autophagosome maturation,^[^ ^45^, ^46^, ^47^, ^48^, ^49^, ^50^ ^]^ we speculated that the ciliary expression of AC3 was required for autophagy regulation, potentially through its interaction with GABARAP (Figure 6L).

### 2.6 Mice with GABARAP KD in the VMH Exhibit Exacerbated HFD‐Induced Obesity

**Figure 7:**
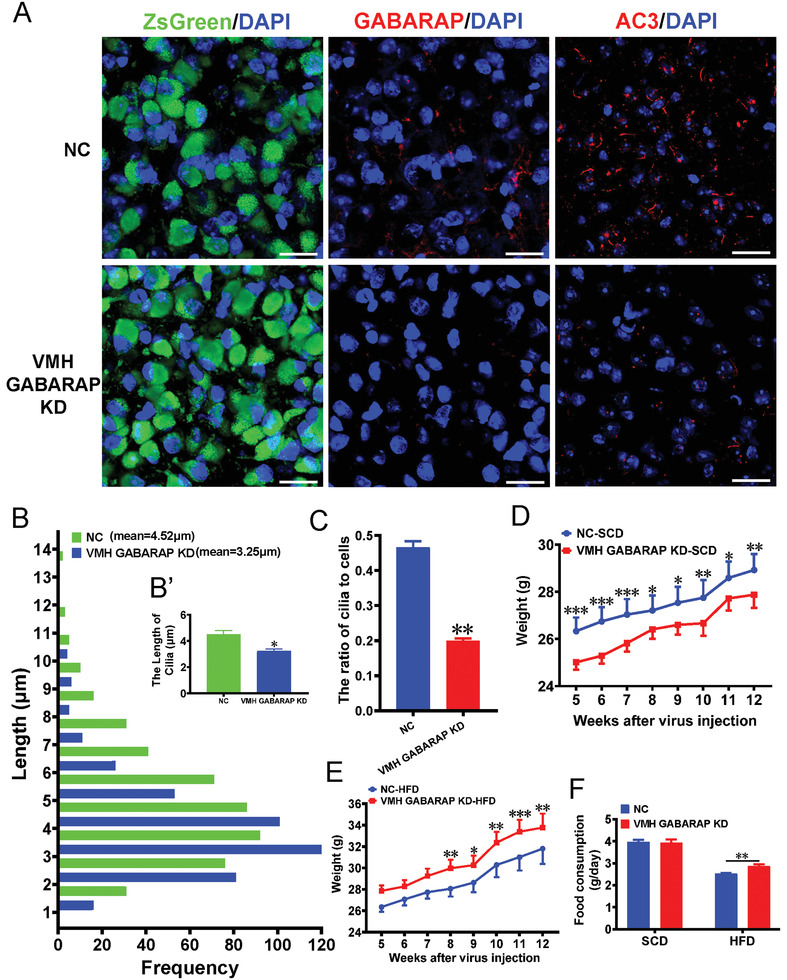
VMH GABARAP KD mice show increased weight under HFD feeding conditions. A) Representative images showing the expression levels of GABARAP and AC3 in the VMHs of VMH GABARAP KD mice and the controls. Scale bars: 20 µm. B) Quantification of the length of cilia in the VMHs of VMH GABARAP KD mice and controls (*n* = 3 mice per group). C) Quantification of the number of cilia in the VMHs of VMH GABARAP KD mice and controls (*n* = 3 mice per group). D, E) The growth curves of NC and VMH GABARAP KD mice starting at 5 weeks after virus injection under SCD feeding (D) and HFD feeding (E) conditions (*n* = 9 mice per group). F) Twenty‐four hours of food consumption in NC and VMH GABARAP KD mice (*n* = 9 mice per group). Data represent the mean ± SEM; ^*^ *p* < 0.05, ^**^ *p* < 0.01, and ^***^ *p* < 0.001; Student's *t*‐test or one‐way ANOVA and Bonferroni pairwise comparisons.

Since ciliary AC3 expression in the VMH was shown to be crucial not only for body weight maintenance but also for autophagic regulation via interaction with GABARAP, it was speculated that the neuronal expression of GABARAP in the VMH may also participate in body weight regulation. To address this possibility, we stereotaxically injected AAVs carrying pMecp2‐spCas9+GABARAP sgRNAs into the VMHs of 4‐week‐old male mice, and pMecp2‐spCas9+NC sgRNA was used as a control (Figure S10A, Supporting Information). GABARAP KD efficiency was ascertained 6 weeks after AAV injection (**Figure** **7**A; Figure S10B,C, Supporting Information). The number of AC3^+^ cilia in the VMH was significantly decreased and the cilia were shorter in the VMHs of VMH GABARAP KD mice compared with NC mice (Figure 7A–C). Furthermore, IF analysis showed that the LC3‐II positive signal was significantly increased in the VMH of GABARAP KD mice compared with their controls (Figure S10D,E, Supporting Information).

The mice were weighed weekly for a period of 12 weeks after AAV injection. Under SCD feeding conditions, the weight curves diverged between the VMH GABARAP KD mice and the controls four weeks after AAV injection (Figure 7D). By the 12th week, the weight of the SCD‐fed NC mice had increased by 49.92%. However, the weight of the SCD‐fed VMH GABARAP KD mice had increased by only 40.31% (Figure 7D). The VMH GABARAP KD mice did not exhibit a significant difference in SCD intake relative to the control mice as of the 12th week after AAV injection (Figure 7F). We also challenged the mice with HFD. In contrast to the SCD feeding results, the VMH GABARAP KD mice showed more pronounced HFD‐induced obesity than the controls (Figure 7E). After only five weeks, the weight of the GABARAP KD mice had increased significantly, whereas the weights of the NC mice did not show a significant increase until week ten of HFD feeding relative to the corresponding SCD‐fed cohorts (Figure S10F, Supporting Information). By the 12th week, the weight of the HFD‐fed NC mice had increased by 73.39% relative to their weight at the time of AAV injection. However, the weight of the HFD‐fed VMH GABARAP KD mice had increased by 81.56% (Figure 7E). Additionally, the HFD intake of the VMH GABARAP KD mice was significantly lower than that of the controls in the 12th week after AAV injection (Figure 7F). Together, these results indicate that GABARAP in the mouse VMH is required for body weight maintenance under HFD feeding conditions.

## 3 Discussion

GWASs and whole‐exome sequencing studies have shown that hAC3 is strongly linked to the body mass index in human populations.^[^ ^19^, ^20^, ^21^, ^22^, ^23^, ^24^, ^25^, ^26^, ^27^ ^]^ However, the underlying mechanisms have not yet been established. Because it is not possible to directly genetically modify hAC3 in the human body, we generated humanized AC3 knock‐in mice by introducing the complete hAC3 sequence into the Rosa26 site in the mouse genome. We further showed that hAC3 mice were not only resistant to HFD‐induced obesity but also showed increases in the length and number of cilia in the VMH. The observations that cilia in the VMH are essential for normal body weight maintenance and neuronal ciliary lengths are selectively reduced in the hypothalami of HFD‐induced obese mice^[^ ^51^ ^]^ strongly suggest that the resistance to HFD‐induced obesity in hAC3 mice could be at least partly attributed to the overexpression of hAC3 in ciliary compartments.

To further verify that the functions of AC3 in modulating body weight are caused via its ciliary expression in the hypothalamic VMH, we specifically knocked down the expression of AC3 in the VMH via a Cas9 AAV stereotactic injection approach. Mice with AC3 KD in the VMH (VMH AC3 KD mice) were susceptible to HFD‐induced obesity, although their weight was not altered under SCD feeding conditions. This mirrored the phenotypes of the hAC3 mice that we generated, which were more resistant to HFD‐induced obesity. We then specifically knocked down ciliary AC3 in the VMH by stereotactically injecting pIFT88‐AC3 shRNA AAV into the mouse VMH to achieve specific AC3 KD in cilia (VMH pIFT88‐AC3 KD mice). Consistent with the findings in VMH AC3 KD mice, VMH pIFT88‐AC3 KD mice were more susceptible to HFD‐induced obesity than the controls, whereas their weights did not differ from those of the controls under SCD feeding conditions. Therefore, our data solidly demonstrate that appropriate AC3 expression in the cilia of the VMH is critical for body weight maintenance, at least under HFD feeding conditions.

Global AC3 knockout mice (AC3^−/−^) are obese under SCD feeding.^[^ ^7^ ^]^ Compared with AC3^−/–^ mice, the body weight phenotypes of VMH AC3 KD or VMH pIFT88‐AC3 KD mice are much less pronounced, and only under HFD feeding conditions do they become obese. The phenotypic discrepancy among these animals is probably due to the following reasons: 1) in addition to VMH, AC3 is also expressed in the cilia of other areas of the brain and peripheral organs;^[^ ^6^, ^52^ ^]^ 2) in addition to neurons, AC3 is also expressed in glial cells;^[^ ^53^ ^]^ 3) ciliary AC3 is developmentally expressed in the brain;^[^ ^6^, ^54^ ^]^ 4) AC3 may function in other compartments of the cell outside of cilia,^[^ ^55^, ^56^ ^]^ all of which might also contribute to body weight regulation.

It was presumed that the specific inhibition of ciliary AC3 expression could be achieved by the exogenous expression of a constitutively active version of GPR88* in targeted neurons.^[^ ^30^ ^]^ Following this hypothesis, we stereotactically injected GPR88*‐packaged AAVs into mouse VMHs. Our data showed that VMH AAV GPR88* mice exhibited increased susceptibility to obesity under either SCD or HFD feeding conditions and that the phenotype was more severe than those of VMH AC3 KD and VMH pIFT88‐AC3 KD mice. The obesity phenotype of VMH AAV GPR88* mice was comparable to that of mice in which GPR88* is exogenously expressed in Sim1‐expressing neurons within the PVN region.^[^ ^30^ ^]^

Recent studies have shown that GPR88 inhibits multiple GPCRs, affecting both their G protein‐ and *β*‐arrestin‐dependent signaling pathways.^[^ ^57^ ^]^ Additionally, GPCRs can promote the formation of G*α*i:*β*‐arrestin complexes that then serve as functional signaling complexes.^[^ ^58^ ^]^ Furthermore, all components of these complexes have been revealed to be localized to cilia and to be involved in diet‐induced obesity regulation.^[^ ^59^, ^60^, ^61^, ^62^, ^63^, ^64^, ^65^, ^66^ ^]^ It is difficult to distinguish the inhibition of ciliary AC3 expression from effects on other signaling pathway components. Thus, we feel that care should be taken in the attribution of the obesity phenotypes induced by exogenous GPR88* expression in MC4R‐expressing neurons of the PVN to the inhibition of ciliary AC3 expression.

The VMH is the region that harbors the longest neuronal cilia,^[^ ^28^, ^29^ ^]^ and the length of VMH primary cilia is dynamically modulated by nutrient conditions.^[^ ^51^ ^]^ Mice in which the VMH neuronal cilia are destroyed^[^ ^28^ ^]^ or the Bbs1 gene is ablated in SF1 neurons within the VMH develop obesity phenotypes.^[^ ^67^ ^]^ At another layer, hypothalamic autophagy has been linked with metabolic regulation.^[^ ^68^ ^]^ In mice, the deletion of the autophagy‐related gene Atg7 specifically in pro‐opiomelanocortin (POMC) or DMH neurons results in metabolic perturbations.^[^ ^14^, ^69^, ^70^, ^71^ ^]^ Similarly, the knockout of Atg12 in mouse POMC neurons induces weight gain,^[^ ^16^ ^]^ whereas mice in which Atg4 is ablated in POMC neurons or Atg7 is ablated in AgRP neurons develop a lean phenotype.^[^ ^13^, ^15^ ^]^AC3 is abundantly enriched in almost all VMH neuronal cilia, and AC3^−/−^ mice develop age‐dependent obesity.^[^ ^7^ ^]^ Given that cilia and autophagy are reciprocally regulated,^[^ ^41^, ^72^, ^73^ ^]^ we speculated that HFD‐induced obesity in mice subjected to ciliary AC3 KD in the VMH might be mediated by autophagy. In the present study, we revealed that autophagic activity was negatively regulated by ciliary AC3 expression, and we further showed that AC3 expressed in cilia may interact with GABARAP through its LIR/GIM site (aa 448‐493) to regulate autophagy in the mouse VMH. Thus, our results might indicate a potential link between ciliary AC3 expression and GABARAP‐associated autophagy in response to HFD‐induced obesity in the mouse VMH.

In addition to its known autophagic roles, the functional effect of GABARAP is also implicated in cytoplasmic vesicle trafficking, such as the trafficking of GABAA receptors in neurons.^[^ ^74^, ^75^, ^76^ ^]^ Accumulating evidence has shown that that most, if not all, ATGs also possess functions independent of their canonical autophagy pathway.^[^ ^77^, ^78^, ^79^, ^80^, ^81^ ^]^ Furthermore, the present knockdown of AC3 expression in VMH GABARAP KD mice was not completely ciliary site specific. Therefore, we cannot rule out potential effects other than cilia and noncanonical autophagy in the phenotype of the more pronounced HFD‐induced obesity of VMH GABARAP KD mice. Accordingly, whether the severe HFD‐induced obesity HFD phenotype determined by AC3 VMH cilia expression was caused by GABARAP‐mediated autophagy requires further study.

It should be noted that the effects of hAC3, AC3‐KD, mGPR88, and GABARAP‐KD on body weight and food consumption in mice fed with SCD or HFD were heterogenous and somewhat varied (Table S1, Supporting Information). We considered these differences to be due to the following reasons. First, the promoters employed in these KD mice were different. For VMH AC3 KD and VMH GABARAP KD mice, we used the Mecp2 promoter, for VMH GPR88 overexpression mice, the Syn2 promoter, and for VMH pIFT88 AC3 KD mice, the IFT88 promoter. Thus, the cell types involved with KD in the VMH might differ, potentially contributing to the discrepancies in the body weight phenotypes in these animal models. Second, the knockdown methods employed in these KD mice were different. For VMH AC3 KD and VMH GABARAP KD mice, knockdown was carried out using the CRISPR/Cas9 approach, for VMH GPR88 overexpression mice, overexpression was carried out by AAV stereotaxic injection, and for VMH pIFT88 AC3 KD mice, knockdown was carried out by the shRNA approach. Therefore, the knockdown efficiency of these animals might be different, which also resulted in the heterogenous body weight and food consumption observed in these mice. For example, global AC3 knockout mice (AC3^−/−^) are obese under SCD feeding,^[^ ^7^ ^]^ whereas an intermediate phenotype was observed in heterozygous null mice (AC3^+/−^) with only HFD feeding conditions without hyperphagia.^[^ ^82^ ^]^

By coupling with G protein *α*‐subunit G*α*s, GPCRs stimulate the activation of adenylyl cyclase to produce intracellular cAMP. Therefore, mice with a deficiency of G*α*s in VMH may demonstrate similar phenotypes to VMH ciliary AC3 KD mice. Somewhat to our surprise, mice carrying a homozygous deletion of G*α*s in SF1‐expressing cells of the VMH (VMHGsKO) showed no alterations in body weight or food intake when raised under SCD or HFD feeding conditions.^[^ ^83^ ^]^ This result might be due to the crosstalk between stimulatory and inhibitory G protein *α* subunits to compensate for the deletion of G*α*s protein.^[^ ^84^, ^85^, ^86^ ^]^ It is also possible that G*α*s and AC3 are distributed in different cell types within the VMH and exert different physiological functions in body weight regulation. For example, GPCR TGR5 couples with G*α*i protein in ciliated cells and G*α*s protein in nonciliated cholangiocytes, respectively.^[^ ^87^ ^]^ It has also be observed that G*α*s is enriched in the primary cilium of granule neuron precursors but is hardly detectable in MEFs.^[^ ^88^ ^]^ Compared with VMH Gs KO mice, mice carrying a homozygous G*α*s deficiency in MC4R‐expressing cells are obese and hyperphagia.^[^ ^89^ ^]^ Nevertheless, it will be interesting to determine whether AC3 and G*α*s are colocalized in the primary cilium within VMH neurons.

## 4 Conclusion

In summary, we showed that ciliary AC3 expression in the VMH interacts with GABARAP‐mediated autophagy in response to high fat‐induced signaling to regulate body weight. Our findings provide insight into how ciliary AC3 functions in the VMH to maintain body weight homeostasis and suggest that AC3 might be a new target for the treatment of obesity.

## 5 Experimental Section

### Experimental Animals

All experimental procedures used in the study followed the Guiding Opinions on the Treatment of Experimental Animals issued by the Ministry of Science and Technology, People's Republic of China, and were approved by the Animal Ethics and Caring Committee of Hebei University (approval no.: IACUC‐2017013). AC3^+/+^ and AC3^−/−^ mice were bred from AC3^+/−^ mice, and the genotype was authenticated with PCR. hAC3 knock‐in mice were generated in the lab (detailed in the Experimental Section in the Supporting Information). C57BL/6N WT mice were purchased from Charles River (Beijing, China). All mice used in this study were male at the age of three months, unless indicated. The mice were housed under a 12 h light (07:00 to 19:00)/dark (19:00 to 07:00) cycle at 22 °C and fed an SCD or HFD (45% fat; Research diet, Rodent Chow #D12451) ad libitum in a specific pathogen‐free (SPF) animal room at Hebei University.

### MEF Cultures

Pregnant AC3^+/−^ and hAC3 mouse dams were sacrificed to harvest their embryos, and the heads, limbs, tails, and internal organs of the embryos were dissected. The remainder of each embryo was then finely minced using a sterile razor blade and placed in a 10 cm round Petri dish in Dulbecco's modified Eagle's (DMEM) supplemented with 10% fetal bovine serum (FBS), 100 U mL^−1^ penicillin, and 10 mg mL^−1^ streptomycin. Cells were grown until reaching ≈80% confluence and were then trypsinized, centrifuged, resuspended in supplemented DMEM, counted, and passaged. Genotypes were analyzed by PCR. All MEFs were cultured in humidified 5% CO2/95% air at 37 °C.

### Cell Cultures

The NIH3T3 and 293T cell lines were maintained in DMEM (Gibco) supplemented with 10% FBS (Gibco) and 1% penicillin/streptomycin (Gibco) in a 37 °C incubator with a humidified 5% CO2 atmosphere.

### DNA Editing Assay of Guide RNAs in NIH3T3 Cells

Optimal guide RNA identification was performed according to reported protocols.^[^ ^90^ ^]^ In brief, the sequence of mouse AC3 exon 1 was used to design guide RNAs with CRISPROR (http://crispor.tefor.net/) to knock down AC3. According to the MIT Specificity Score, 4–5 sgRNAs were synthesized in BGI (Beijing, China). The guide RNAs were cloned into pX459 (Addgene, #48139). Subsequently, the recombinant plasmids were transfected into NIH3T3 cells, and the transfected cells were successfully screened with hypoxanthamycin. Genomic DNA was extracted, and the targeted fragments were amplified by PCR. The PCR products were digested by using T7E1, and insertion/deletion (InDel) rates were calculated. The guide RNA with the highest InDel rate was selected as the optimum guide RNA referring to GABARAP sgRNAs.^[^ ^91^ ^]^

### Vectors and Virus Production

To build lentiviral vectors to knock down AC3 in the cilia of MEFs, the target sequence 5′‐GCAGATATTGTGGGCTTTA‐3′ for AC3 was shuttled in the pLKO.1‐hygromycin vector (Addgene, #24150). The mouse IFT88 promoter was employed as the shAC3’ promoter. Viral particles were packaged into Lenti‐X 293T cells (Takara bio) and cotransfected with two packaging plasmids, pCMV‐VSV‐G (Addgene, #8454) and pCMV‐dR8.2 dvpr (Addgene, #8455). Viral particles were concentrated by ultracentrifugation in a Beckman XL‐90 centrifuge with an SW‐28 rotor at 20, 000 rpm for 120 min at 4 °C.

The spCas9 and guide RNA expression cassettes were separately packaged into two AAVs. The short neuron‐specific promoter of Mecp2 was used to drive the expression of spCas9. The spCas9 sequences were cloned into AAV2/9 vectors in tandem with the pMecp2 sequence in a construct referred to as spCas9 AAV. The AC3 guide RNA, GABARAPL guide RNA, and NC guide RNA were cloned into the AAV2/9 vector in tandem with the U6 promoter and pSyn2‐mCherry (reporter gene) to generate the sgRNA AAVs.

To construct AAV vectors for AC3 KD in the cilia of the mouse VMH, the mouse AC3 target sequence 5′‐GCAGATATTGTGGGCTTTA‐3′ and the IFT88 promoter sequence were inserted into the pHBAAV‐hSyn‐ZsGreen vector. To specifically express GPR88* in neurons, the Syn2 (a brain neuron‐specific gene) promotor was used to drive the expression of GPR88*. A fragment containing the GPR88* CDS and the GFP CDS was cloned into the pHBAAV‐hSyn promoter vector.

Viral particles of AAV2/9 were packaged in HEK293T cells with two other plasmids: pAAV‐RC and pAAV‐Helper (Hanbio, Shanghai). HEK293T cells were harvested 72 h post‐transfection by scraping and pelleting the cells via centrifugation. The viral particles were purified with a heparin column (GE Healthcare) and then concentrated with an Ultra4 centrifugal filter unit (Amicon, 100, 000 molecular weight cutoff). The titers of viral particles were determined by quantitative PCR to obtain >1 × 10^12^ particles mL^−1^.

### Lentivirus Transfection into MEFs

To obtain IFT88‐AC3‐KD MEFs, LV‐pIFT88‐shAC3‐pCMV‐ZsGreen was transfected into WT MEFs. LV‐pIFT88‐shNC‐pCMV‐ZsGreen was used as a negative control. Three days post‐transfection, the MEFs were processed for IF with antibodies against AC3 or ARL13B, and their lysates were isolated for Western blot analysis with antibodies against AC3 or ARL13B, while an antibody against actin was used as the control.

### Stereotactic Injection of AAV into the Mouse VMH

To generate VMH NC, AC3 KD, and GABARAP KD mice, weight‐paired male mice at 4 weeks old were lightly anesthetized with isoflurane and injected with 500 nL of a 1:1 AAV mixture (1.1 × 10^12^ vector genomes (Vg) mL^−1^) of pMecp2‐SpCas9; 1.1 × 10^12^ Vg mL^−1^ of AC3 sgRNA or GABARAP sgRNA or NC sgRNA) at each site in the VMH (*x* = ±0.5, *y* = −1.4, and *z* = −5.6).

To generate VMH AAV GPR88* mice and the corresponding controls, male mice (4 weeks old) were lightly anesthetized with isoflurane, and 500 nL of pSyn2‐GPR88* AAV (3.5 × 10^12^ Vg mL^−1^) or NC AAV was injected at each site in the VMH (*x* = ±0.5, *y* = −1.4, and *z* = −5.6). Similarly, pIFT88‐shAC3 AAV (3.5 × 10^12^ Vg mL^−1^) or NC AAV (500 nL) was injected into the VMH to generate a mouse model of specific AC3 KD in VMH cilia.

### Body Weight and Food Intake Measurements

After AAV injection, the mice in each treatment group were divided into two groups, one of which was fed a normal diet, while the other was fed an HFD (45% fat; Research diet, Rodent Chow; Catalog #D12451), and the weight of each mouse was measured regularly every week. For the measurement of the daily food intake, the experimental mice were fed separately, and their food intake was measured daily at 8 am for seven consecutive days. The average daily food intake was then calculated.

### Metabolic Parameter Measurements

Mice were maintained in a comprehensive laboratory animal monitoring system (CLAMS; Columbus Instruments, Columbus, OH) for 24 h to adapt them to this environment. The O2 consumption volume, CO2 production volume, heat production, total activity, and food intake were continuously recorded during the next 48 h according to the instructions of the manufacturer. The RER was calculated according to the formula RER = VCO2/VO2.

### Serum Hormone Content Measurements

The mice were fasted for 12 h, and blood was then collected from the mouse tail. The concentrations of hormones in the sera from each treatment group were measured by ELISA.

### RNA Isolation and qPCR Analyses

Freshly isolated brains were microdissected on ice to isolate the hypothalamus. Total RNA was extracted using the TRIzol method, and first‐strand cDNA was synthesized using a Universal RiboClone cDNA Synthesis System (Promega; Catalog # C4360). mRNA expression was assessed with NovaTM SYBR Green PCR Master Green mix (Qiagen; Catalog # 204143), and analysis was performed using the 2‐${}^{\wedge \wedge }$CT method. The results for all qPCR samples were normalized to actin.

### Western Blot Assay

Protein was extracted from hypothalamus tissues or MEFs using RIPA lysis and extraction buffer (Pierce; Catalog # 89900), and the protein concentration was determined using the bicinchoninic acid (BCA) method. Equal amounts of protein from each sample were separated by SDS‐polyacrylamide gel electrophoresis, and the separated proteins were then transferred to Immobilon‐PSQ Transfer Membranes (Millipore). The membranes were incubated in blocking solution (5% nonfat dry milk in TBS) at room temperature (RT) for 1–2 h. Incubation with the primary antibodies [including antibodies against AC3 (1:500; Invitrogen; Catalog # PA5‐35382), ARL13B (1:1000; Proteintech; Catalog # 17711‐1‐AP), LC3B (1:500; Sigma‐Aldrich; Catalog # L7543), SQSTM1/p62 (1:1, 1000; Abcam; Catalog # ab56416), GABARAP (1:2, 000; Abcam; Catalog # ab109364), ATG5 (1:500; Proteintech; Catalog # 10181‐2‐AP), VPS34 (1:500; Novus; Catalog # NB110‐87320), Beclin1 (1:500; Novus; Catalog #NB500‐249), GAPDH (1:2000; Proteintech; Catalog # 60004‐1‐Ig), and actin (1:2000; Proteintech; Catalog # 60009‐1‐lg)] was performed at 4 °C overnight. Incubation with the secondary antibody (conjugated with 680 or 800 nm fluorophores, SeraCare KPL, 1:10 000 in TBST buffer) was conducted for 1–2 h at RT. Images were obtained using Odyssey software (Li‐Cor). The gray value of the protein of interest was quantified by using ImageJ, and the relative expression level of the protein was estimated as the gray value of the protein of interest/the gray value of the loading control. GAPDH and actin were used as the loading controls.

### No‐Serum Treatment of MEFs

To induce autophagy, AC3^−/−^, AC3^+/+^, hAC3 MEFs, and NC and pIFT88‐AC3 KD MEFs were maintained in DMEM (Gibco) supplemented with 1% penicillin/streptomycin (Gibco). MEFs maintained in DMEM supplemented with 10% fetal bovine serum and 1% penicillin/streptomycin were used as negative controls. Twenty‐four hours later, the MEFs were processed for the isolation of lysates, which were then analyzed by Western blot with antibodies against LC3B and p62; actin was used as the loading control.

### Autophagy Inhibitor/Inducer Treatment of the MEFs

3‐MA (Sigma‐Aldrich; Catalog # 189490, 100 nmol), rapamycin (Sigma‐Aldrich; Catalog # V900930; 5 µmol), and bafilomycin A1 (Sigma‐Aldrich; Catalog # 19‐148, 100 nmol) were separately added to AC3^−/−^, AC3^+/+^, and hAC3 MEFs maintained in DMEM (Gibco) supplemented with 10% fetal bovine serum (Gibco) and 1% penicillin/streptomycin (Gibco). Three hours later, the MEFs were processed for the isolation of lysates, which were then analyzed by Western blot with an antibody against LC3B, while actin was used as the loading control.

### Autophagic Flux Assays

Autophagic flux was tested by the transient transfection of the mCherry–GFP–LC3 plasmid into AC3^+/+^, AC3^−/−^, and hAC3 MEFs. Constructs were transfected using Lipofectamine 3000 reagent (Invitrogen; Catalog # L3000150) according to the manufacturer's instructions. The MEFs were treated in a serum‐free manner for 3 h. Fluorescence signals were observed using an Olympus FLUOVIEW FV3000 confocal microscope. The quantification of the yellow and red puncta was performed with the Green and Red Puncta Colocalization Macro for ImageJ.

### Transmission Electron Microscopy and Morphometric Analysis

AC3^+/+^, AC3^−/−^, and hAC3 MEFs grown to confluence were pelleted and fixed in 2.5% glutaraldehyde in 100 × 10^−3^ m sodium cacodylate (SC, pH 7.43) for 45 min at RT. The pellets were then rinsed in SC, postfixed in 1% osmium tetroxide in SC, treated with 1% uranyl acetate, dehydrated through a graded ethanol series, and embedded in LX112 resin (LADD Research Industries). Ultrathin sections were cut with an ultramicrotome (Lecia UC‐7) and stained with uranyl acetate followed by lead citrate. All grids were viewed on a Hitachi H‐7000 transmission electron microscope at 80 kV. The morphometric analysis of transmitted electron micrographs was performed using ImageJ.

### Tissue Processing and IF

Hypothalamus tissues were fixed in a 4% paraformaldehyde (PFA) solution. After 24 h, the tissues were washed with PBS and dehydrated using 30% sucrose until the tissues were completely covered. The tissues were embedded in OCT medium (SAKURA) and cut into 30 µm thick sections using a cryostat.

The tissue sections were washed for 5 min with PBS, permeabilized for 30 min with 0.5% Triton X‐100 in PBS (PBST), blocked with blocking solution (5% donkey serum in PBST solution) for 2 h, and then incubated in primary antibody solution (specific antibody in blocking solution) overnight at 4 °C. Primary antibodies were used against the following antigens: AC3 (1:1000; Novus, Catalog # NBP1‐92683), GABARAP (1:500; Santa Cruz Biotechnology; Catalog # sc‐377300), and LC3B (1:500; Sigma‐Aldrich; Catalog # L7543). Incubation with the secondary antibodies (Alexa 488‐, 594‐, or 568‐conjugated; 1:500; Thermo Scientific) was conducted at RT for 2 h, followed by nuclear counterstaining with DAPI (Sigma; Catalog # D9542). Fluorescent confocal slices were observed using an Olympus FLUOVIEW FV3000 confocal microscope.

For MEF IF, MEFs were washed for 5 min with PBS, fixed for 20 min in a 4% PFA solution, permeabilized for 30 min with 0.5% PBST, blocked with blocking solution (5% donkey serum in PBST solution) for 2 h, and then incubated in the primary antibody solution (specific antibody in blocking solution) overnight at 4 °C. Primary antibodies were used against the following antigens: AC3 (1:1000; Novus, Catalog # NBP1‐92683), ARL13B (1:1000; Neuromab; Catalog # 75‐287), and GABARAP (1:500; Santa Cruz Biotechnology; Catalog # sc‐377300). Incubation with the secondary antibodies (Alexa 488‐, 594‐, or 568‐conjugated; 1:500; Thermo Scientific) was performed at RT for 2 h, followed by nuclear counterstaining with DAPI. Fluorescent confocal slices were obtained using an Olympus FLUOVIEW FV3000 confocal microscope.

### Co‐IP

The CDS of AC3 was amplified from mouse brain cDNA and inserted into the eukaryotic expression plasmid pCMV‐ZsGreen‐N1. The CDS of GABARAP was amplified from mouse brain cDNA and inserted into the eukaryotic expression plasmid pCMV‐3HA‐3Flag. AC3 CDSs with mutated SUMO sites (A: K296R and B: K465R) or mutated LIR sites (A: 488‐493 aa; B: 958‐963 aa) were generated by site‐directed mutagenesis. A 3 µg mixture of the GABARAP CDS and the AC3 CDS or AC3 CDS with mutated SUMO sites or AC3 CDS with mutated LIR sites (1:1) was transfected into NIH3T3 cells using Lipo3000 Transfection Reagent (Invitrogen, Catalog # L3000150). The transfected cells were harvested 72 h after transfection. Protein was isolated in immunoprecipitation buffer (Thermo Scientific, Catalog # 87788) containing a protease and phosphatase inhibitor cocktail (Cell Signaling Technology, Catalog # 7012). The protein lysates were precleared with mouse IgG (Sepharose Bead Conjugate; Cell Signaling Technology; Catalog # 2729) and immunoprecipitated with constant rotation at 4 °C for 16 h using 5 µg of the anti‐AC3 primary antibody (Abcam; Catalog # ab14778) or 5 µg of GFP Tag monoclonal antibody mouse IgG as a negative control (Proteintech; Catalog # 66002‐1‐Ig), followed by a 4 h incubation with mouse IgG (Sepharose Bead Conjugate; Cell Signaling Technology; Catalog # 2729). The samples were washed at least five times on ice using immunoprecipitation buffer with constant rotation, and the beads were then resuspended in 2× SDS loading buffer by boiling and analyzed by Western blot with antibodies against DYKDDDDK Tag (Binds To FLAG Tag Epitope) (Proteintech; Catalog # 66008‐3‐Ig) and GFP (Proteintech; Catalog # 66002‐1‐Ig), while the loading control was performed with an antibody against actin.

### Pull‐Down Assay

The GABARAP CDS was inserted into the prokaryotic expression plasmid pET‐28a. The pET28a‐GABARAP CDS was transfected into BL21(DE3) competent cells. Then, GST‐GABARAP‐overexpressing BL21(DE3) competent cells were scratched, lysed, and sonicated. After centrifugation, 50 µL of the supernatant was retained as the input, and the remaining portion was incubated with a GST Tag Monoclonal Antibody (Proteintech; Catalog # 66001‐2‐Ig) overnight at 4 °C. The mixture was then washed, and protein was isolated to detect the expression of AC3 and GABARAP by Western blot with antibodies against AC3 and GABARAP.

### Statistical Analysis

Statistical comparisons were performed on data originating from at least three biologically independent experimental replicates (as indicated in the figure legends). GraphPad Prism and SPSS 21.0 software were used to conduct the statistical comparisons. For comparisons between two treatment groups, Student's *t*‐test was used. For comparisons among three or more treatment groups, one‐way ANOVA followed by the Bonferroni multiple comparison test was used. The results are shown as the mean ± standard error of the mean (SEM). ^*^ *p*‐values lower than 0.05 were considered statistically significant.

## Conflict of Interest

The authors declare no conflict of interest.

## Author Contributions

D.Y. and X.W. contributed equally to this work. Z.W. and D.Y. designed the experiments. D.Y., X.W., and W.W. performed the experiments. D.Y., X.W., and Y.Z. analyzed the data. All authors discussed the results, and Z.W. and D.Y. wrote the manuscript. All authors read and approved the final version of the manuscript.

## Supporting information

Supporting Information

## Data Availability

Research data are not shared.
