# Supplementary material for: Ciliary Type III Adenylyl Cyclase in the VMH Is Crucial for High‐Fat Diet‐Induced Obesity Mediated by Autophagy
Source: Adv Sci (Weinh). 2021 Nov 16;9(3):2102568. doi: 10.1002/advs.202102568 (PMC8787410; doi:10.1002/advs.202102568)
Supplement: Supplementary file 1 — Supporting Information [file ADVS-9-2102568-s001.pdf]

WILEY-VCH

ADVANCED SCIENCE
Open Access

Supporting Information

for *Adv. Sci.*, DOI: 10.1002/advs.202102568

Ciliary type III adenylyl cyclase in the VMH is crucial for high-fat diet-induced obesity mediated by autophagy

*Dong Yang,† Xiangbo Wu,† Weina Wang, Yanfen Zhou, and Zhenshan Wang**

Supporting Information

Ciliary type III adenylyl cyclase in the VMH is crucial for high-fat diet-induced obesity mediated by autophagy

Dong Yang,[†] *Xiangbo Wu,*[†] *Weina Wang, Yanfen Zhou, and Zhenshan Wang*[*]

Supporting Information Method

Generation of hAC3 knock-in mice

To recombine the hAC3 gene at the Rosa26 site in the mouse genome, the Rosa26 3' and Rosa26 5' homologous arms were amplified by PCR from mouse genomic DNA, and the neogene was obtained from pZsGreen-N1. The BAC Clone CTD-2259I17 [containing a 119.181 kb insert from Chromosome 2 (25,016,005 bp to 25,146,661 bp) (Human Genome Feb. 2009 GRCh37/hg19) with AC3] was modified by red/ET recombination (Counter-Selection BAC Modification Kit; Gene Bridges; Catalog # K002) to insert a Rosa26 3' homologous arm (2.6 kb) into the upstream sequence of AC3 and a neosequence and a Rosa26 5' homologous arm (7.5 kb) into the downstream sequence of AC3. The final BAC construct was purified with a Nucleobond[R] max100 BAC extraction kit (MACHERERY-NAGEL; Catalog # 740579) and verified by sequencing and restriction enzyme mapping.

The BAC constructs were linearized with I-SceI and then electroporated into mouse embryonic stem (ES) cells using a BTX ECM 630 Electro cell manipulator (voltage, 190 V; capacitance, 500 μF; resistance none; BTX, San Diego, CA, USA). ESC clones were selected in hypoxanthine aminopterin thymidine (HAT) and G418 media and isolated, and their DNA was purified. To screen positive ESC clones and verify the integrity of the hAC3 gene inserted into the Rosa26 site of the mouse genome, seven specific PCR primers were designed to span the entire AC3 BAC construct (with a maximum of 28 kb between them and an average distance

of 14.28 kb), and PCR assays were performed. Correctly targeted ESC clones were microinjected into mixed (C57BL/6N and 129) mouse blastocysts to generate chimeras. Male germline offspring were then bred to C57BL/6N females, and backcrossing with C57BL/6N mice was continued until stable staining was observed. The genotype was authenticated using PCR. PCR combined with Sanger sequencing and fluorescence in situ hybridization (FISH) were used to verify the successful insertion of human AC3 into the Rosa26 site in the mouse genome. The expression of AC3 was detected by WB. The hAC3 mice used in this study were N5 or higher.

Supplementary information Table

Supplementary Table 1 The summarized effect of hAC3, AC3-KD, mGPR88, GABARAP-KD on the body weight and the food consumption of mice fed with SCD or HFD, compared to their controls.

Mice	The promoter used to create the mouse line	The method used to create the mouse line	SCD		HFD	
			Body weight	Food Intake	Body weight	Food Intake
**hAC3**	N.A.	BAC homologous recombination	No difference	No difference	Decreased	Increased
**VMH AC3 KD**	pMecp2	CRISPR/Cas9 AAV	No difference	No difference	Increased	No difference
**VMH GPR88**	pSyn2	Overexpression AAV	Increased	Increased	Increased	No difference
**VMH IFT88**	pIFT88	RNAi AAV	No difference	No difference	Increased	Increased
**VMH GAB KD**	pMecp2	CRISPR/Cas9 AAV	No difference	No difference	Increased	Increased

Supporting Information figures

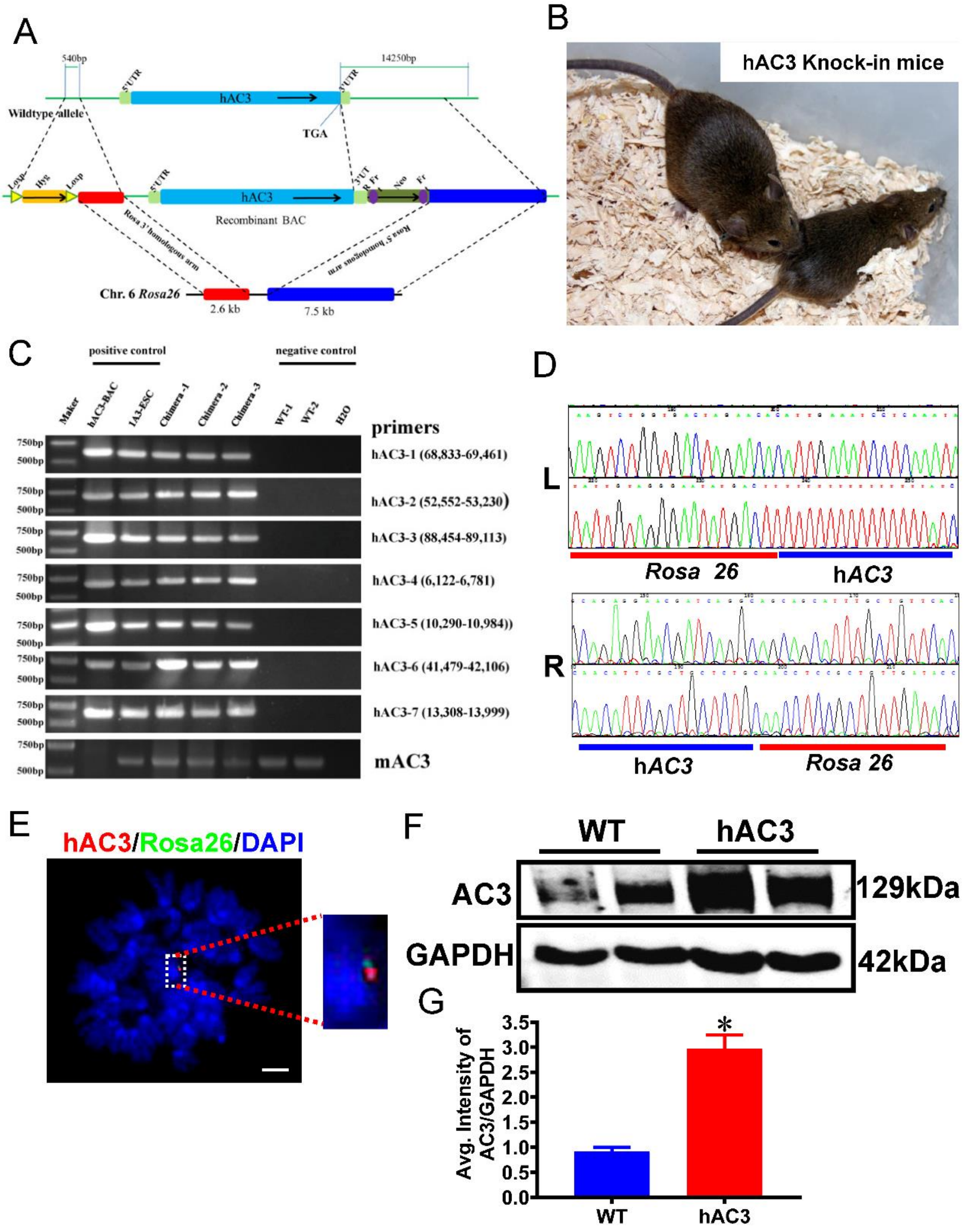

Figure S1. Generation of hAC3 knock-in mice. A) Schematic illustration of the BAC structure containing the hAC3 gene. B) Representative image of hAC3 mice. C) PCR was used to verify the successful construction of the hAC3 mice. The sequence of the hAC3 gene was used as a template, and 8 pairs of primers were designed at different positions in the sequence. hAC3 BAC DNA and validated embryonic stem cell DNA were used as positive controls, and wild-

type mice and H2O were used as negative controls. PCR was used to verify whether the construction of the hAC3 mice was successful. D) Sanger sequencing analysis of the hAC3 gene introduced into the mouse Rosa26 site. The primers were designed to target the insertion site of the Rosa26 gene (the upstream primer targeted the Rosa26 sequence, and the downstream primer targeted the hAC3 sequence). The DNA of the hAC3 mice was used as a template for PCR, and the PCR products were sequenced to determine whether the hAC3 gene was appropriately targeted for knock in at the mouse Rosa26 site. E) FISH analysis of the hAC3 gene targeted to the mouse Rosa26 site. Scale bars, 5 μm. F) WB of AC3 expression in the MOE of hAC3 mice. GAPDH served as the loading control.

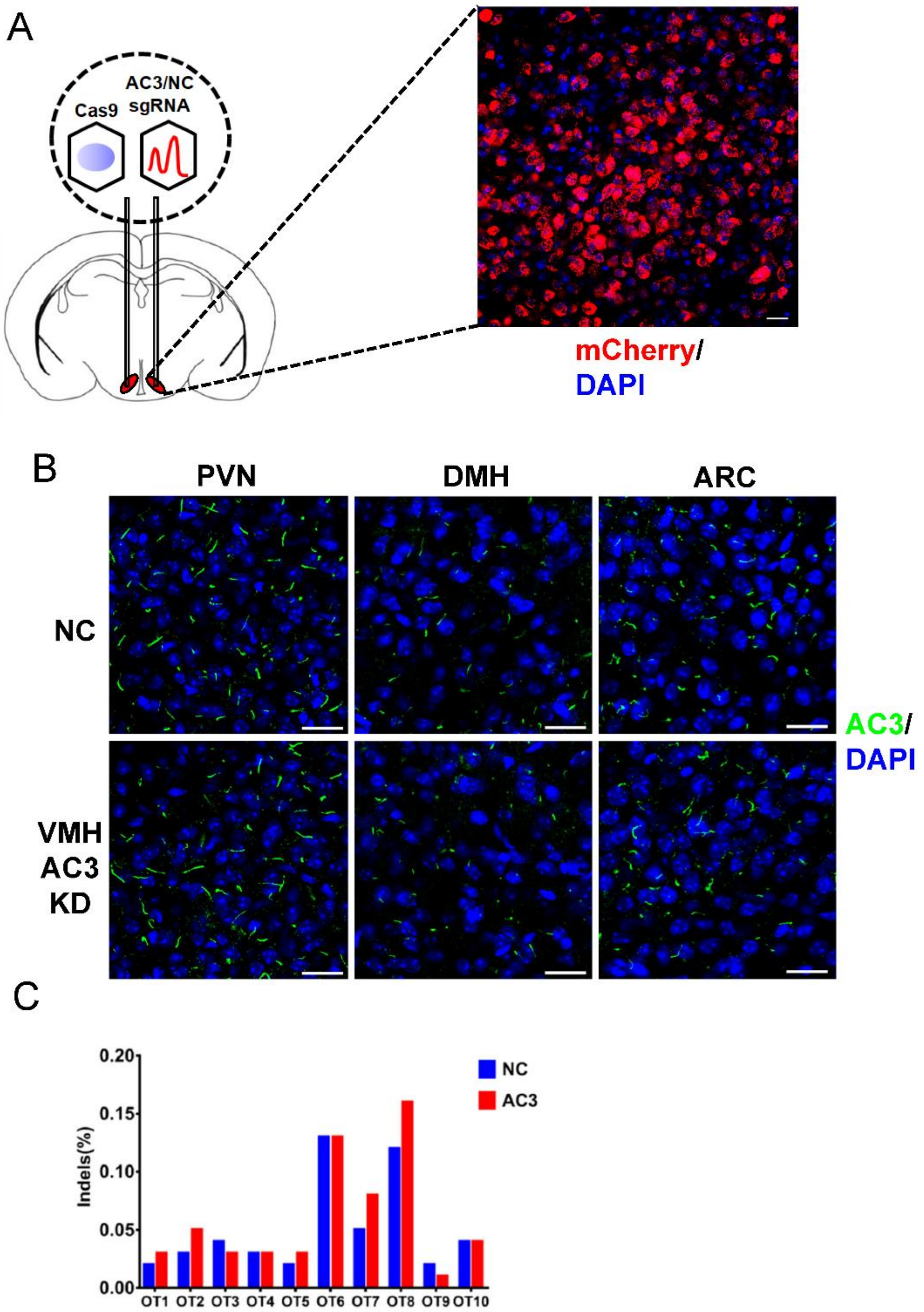

Figure S2. AC3 sgRNA-Syn2-mCherry expression in the VMH, but not in the DMH, ARC or PVN, and off-target analysis of AC3 sgRNA in the VMH. A) Schematic representation of the stereotactic injection of spCas9 and AC3 sgRNA AAV into the mouse VMH and the infection efficiency of the AC3 sgRNA-Syn2-mCherry AAV in the VMHs of VMH AC3 KD mice. B) Representative images showing the expression level of AC3 in the DMH, ARC and PVN of VMH AC3 KD mice and the controls. Scale bars, 20 µm. C) Sequencing assays showing no off-target effects in the hypothalami of VMH AC3 KD mice.

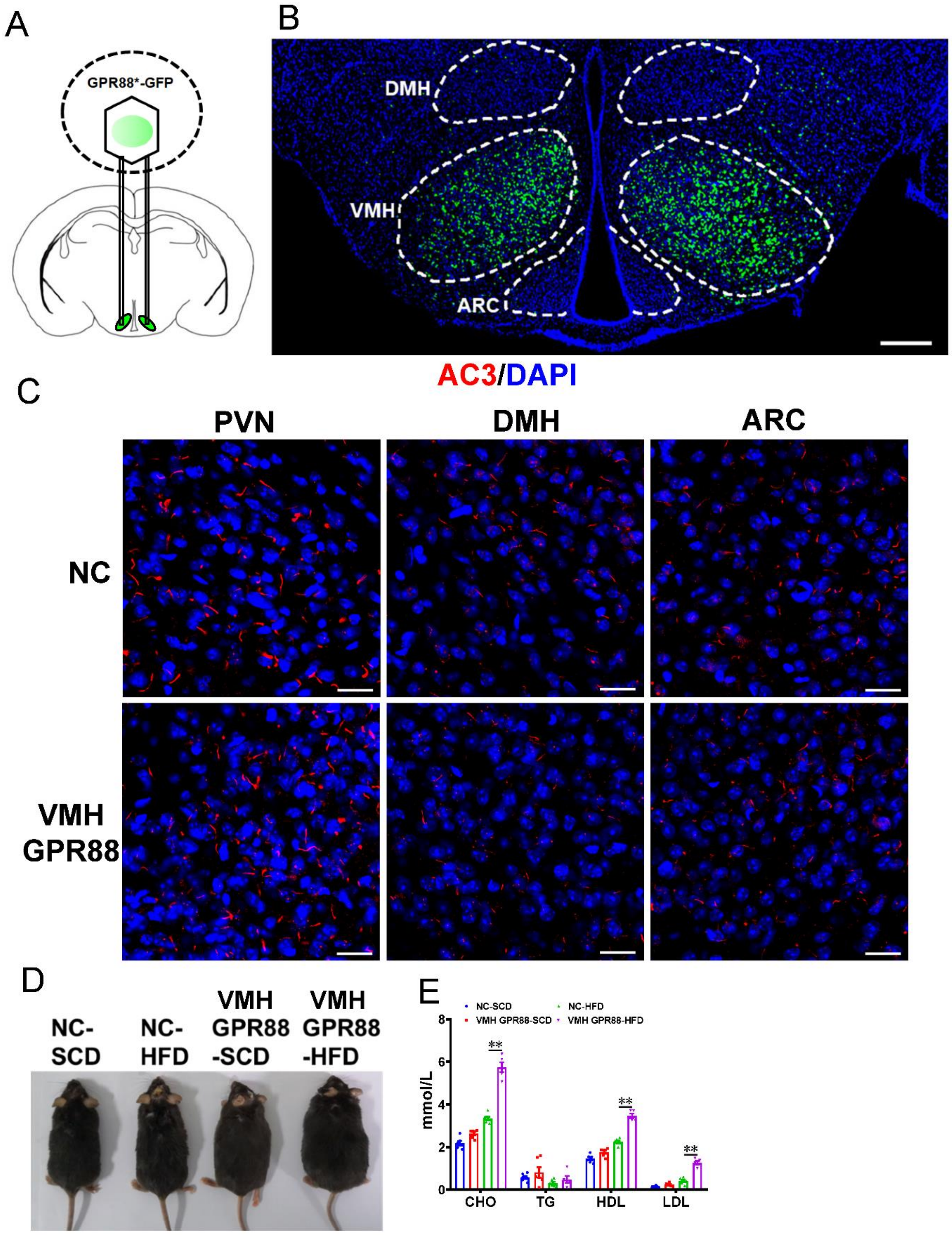

Figure S3. VMH GPR88 mice show obesity. A) Schematic representation of the VMH injection of pSyn2-GPR88*-GFP AAV or pSyn2-GFP AAV. B) Almost all positive GFP signals occur in the VMH. C) Representative images showing the expression of AC3 in the DMH, ARC and PVN of the VMH AAV GPR88* mice and the controls. Scale bars, 20 μm. D)

Representative image of VMH AAV GPR88* mice and controls 8 weeks after AAV injection. E) Hormone levels in the serum of VMH AAV GPR88* mice and controls (n=8 mice per group). Data represent the mean±SEM; **$p<0.01$; One-way ANOVA and Bonferroni pairwise comparisons.

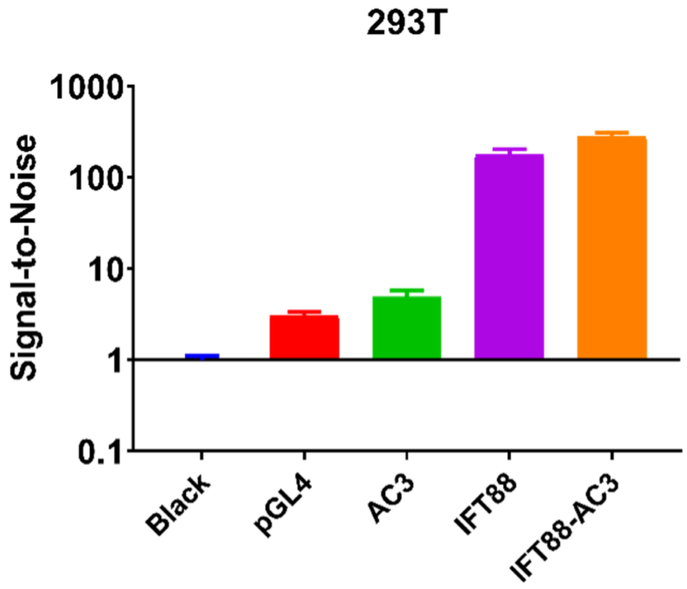

Figure S4. Activity of the IFT88 promoter. The IFT88 promoter fragment was cloned into pGL4.0. The recombinant plasmid was transfected into 293T cells. Luciferase activity was detected 24 h post-transfection. pGL4.0 and AC3 were used as negative controls (n=6 repetitions per group; data represent the mean ± SEM).

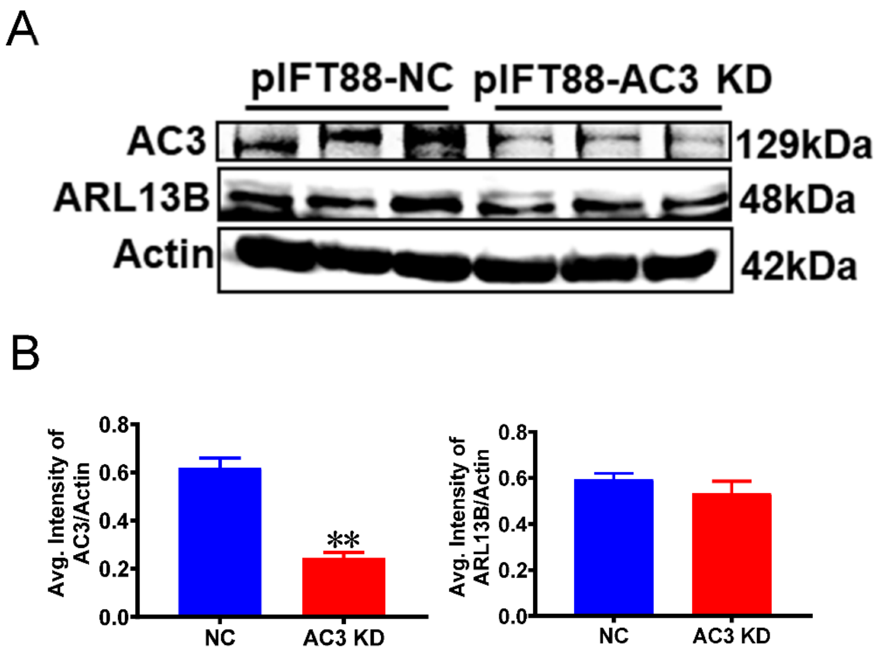

Figure S5. AC3 in cilia was knocked-down in MEFs. A and B) WB (A) and densitometric quantification (B) of AC3 and ARL13B expression in MEFs transfected with LV-pIFT88-shAC3 and controls. Actin served as a loading control (n = 3 repetitions per group).

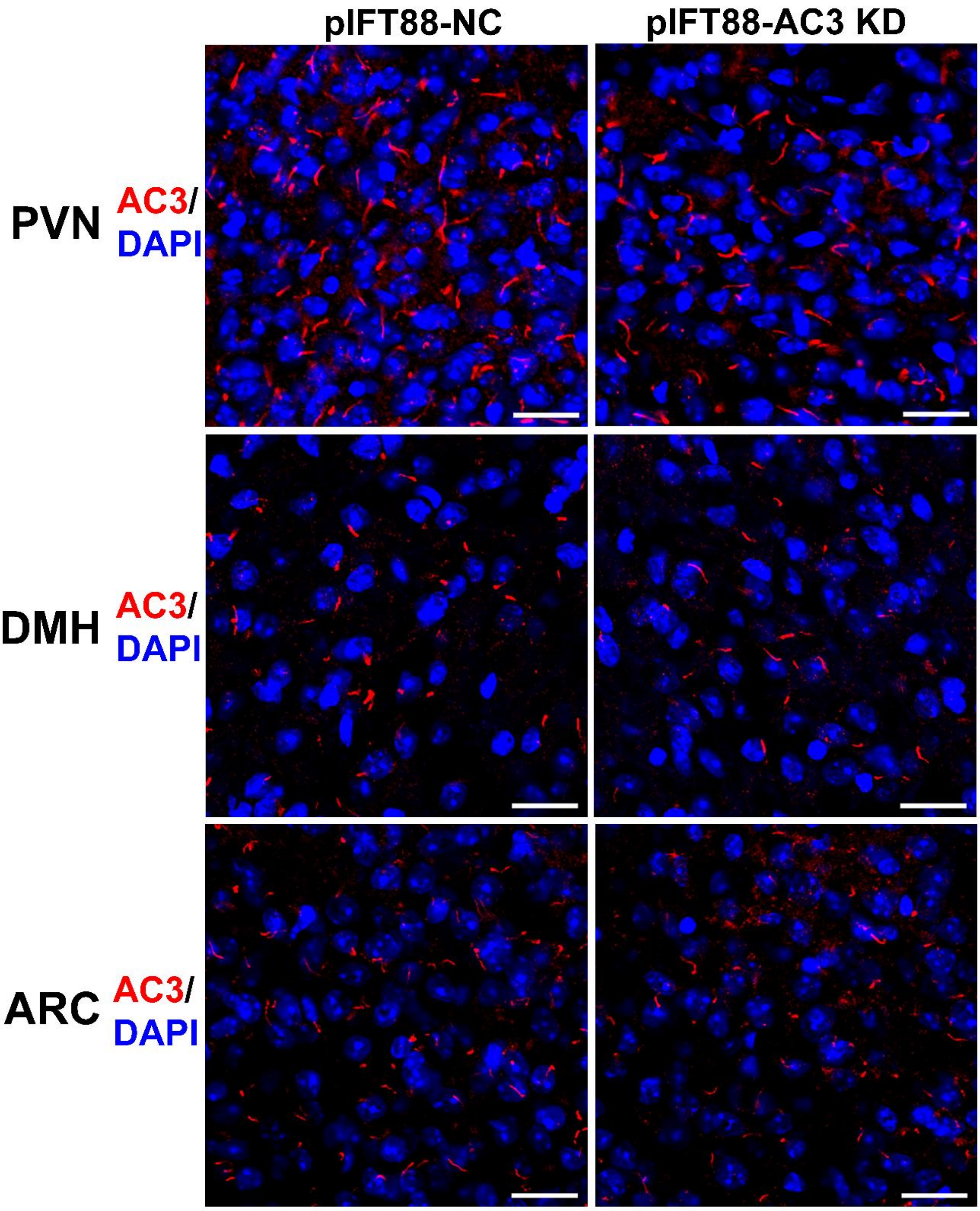

Figure S6. AC3 expression in the PVN, DMH and ARC of VMH pIFT88-AC3 KD mice and the corresponding controls. Representative images showing the expression of AC3 in the DMH, ARC and PVN of VMH pIFT88-AC3 KD mice and the controls. Scale bars, 20 µm.

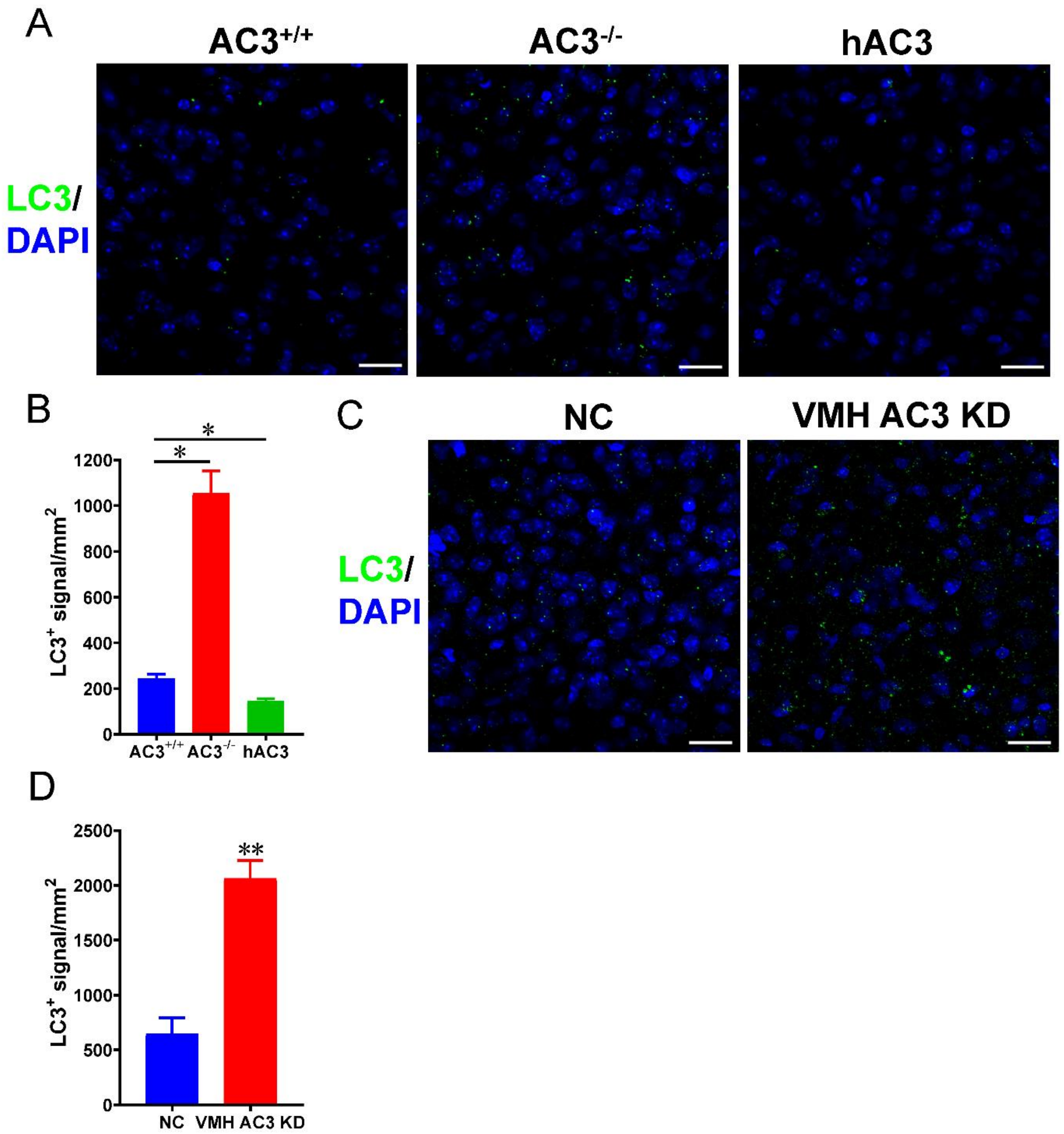

Figure S7. Autophagy levels in the VMH of AC3$^{+/+}$, AC3$^{-/-}$, hAC3, and VMH AC3 KD mice and their controls. A) Representative images showing the expression levels of LC3 in the VMHs of AC3$^{+/+}$, AC3$^{-/-}$, and hAC3 mice. Scale bars, 20 μm. B) Quantification of positive LC3 signals in the VMHs of AC3$^{+/+}$, AC3$^{-/-}$, and hAC3 mice (n=3 mice per group). C) Representative images showing the expression levels of LC3 in the VMHs of VMH AC3 KD mice and their controls. Scale bars, 20 μm. D) Quantification of positive LC3 signals in the VMHs of VMH AC3 KD mice and their controls (n=3 mice per group). Data represent the mean ± SEM; *$p<0.05$, **$p<0.01$; Student's t-test.

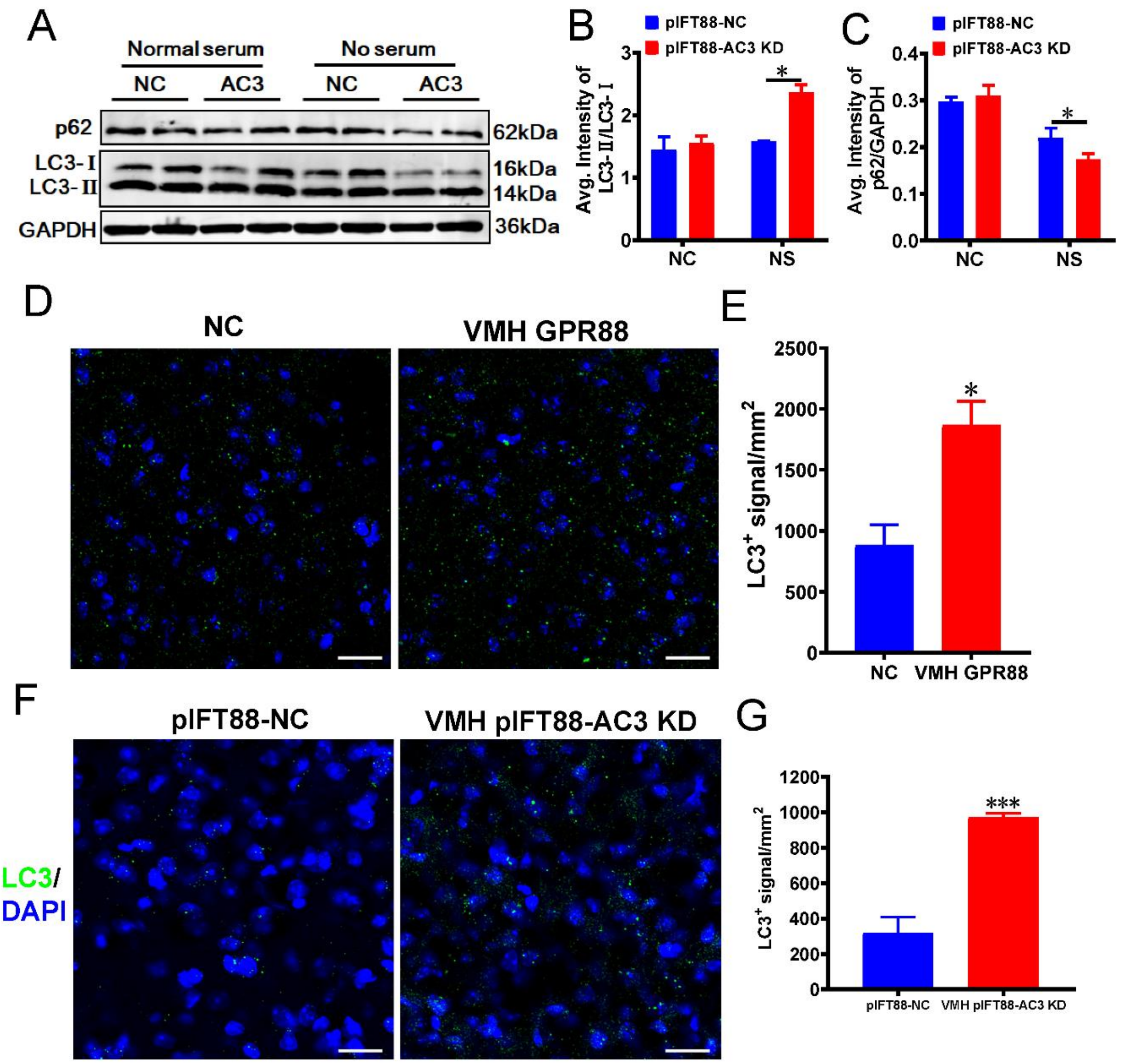

Figure S8. Autophagy levels in ciliary AC3 KD MEFs and VMH. A-C) WB (A) and densitometric quantification of the expression of LC3 (B) and p62 (C) in MEFs transfecting LV-pIFT88-shAC3 and the controls with or without serum addition (n=3 repetitions per group). Actin served as the loading control. D) Representative images showing the expression levels of LC3 in the VMHs of VMH pIFT88-AC3 KD mice and the controls. Scale bars, 20 µm. E) Quantification of positive LC3 signals in the VMHs of VMH pIFT88-AC3 KD mice and the controls (n=4 mice per group). Data represent the mean ± SEM; *p<0.05, ***p<0.001; Student's t-test.

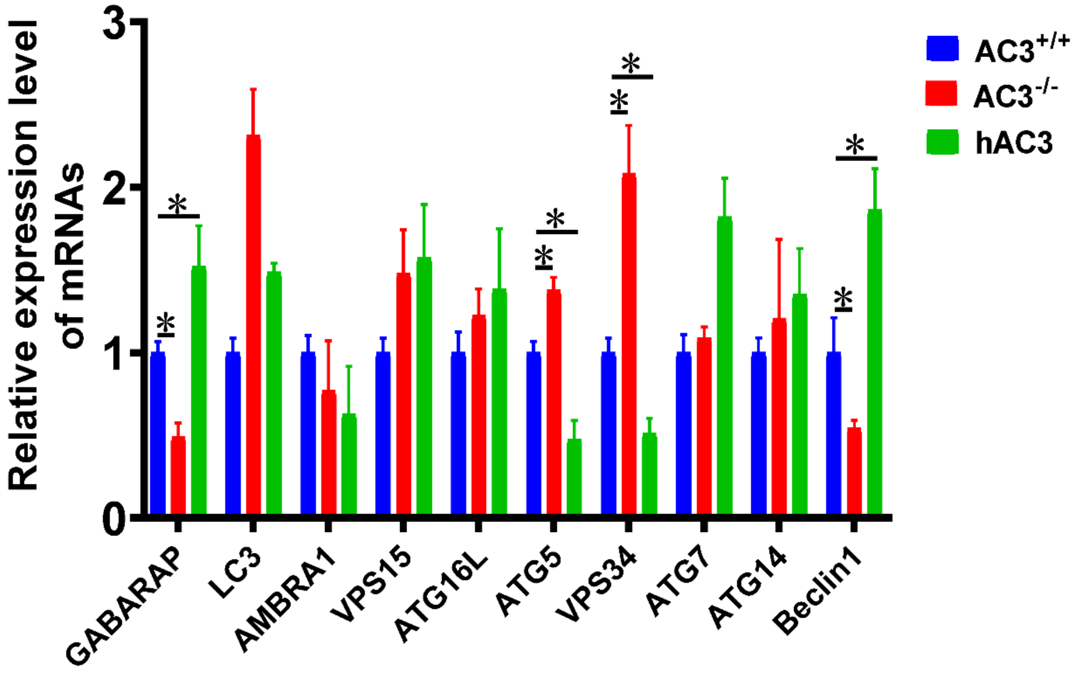

Figure S9. Relative expression levels of reported ATGs in the hypothalami of $AC3^{+/+}$, $AC3^{-/-}$ and hAC3 mice. qPCR analysis of reported ciliary ATGs in the hypothalami of $AC3^{+/+}$, $AC3^{-/-}$ and hAC3 mice (n = 4 mice per group). Data represent the mean±SEM; *$p<0.05$; one-way ANOVA and Bonferroni pairwise comparisons.

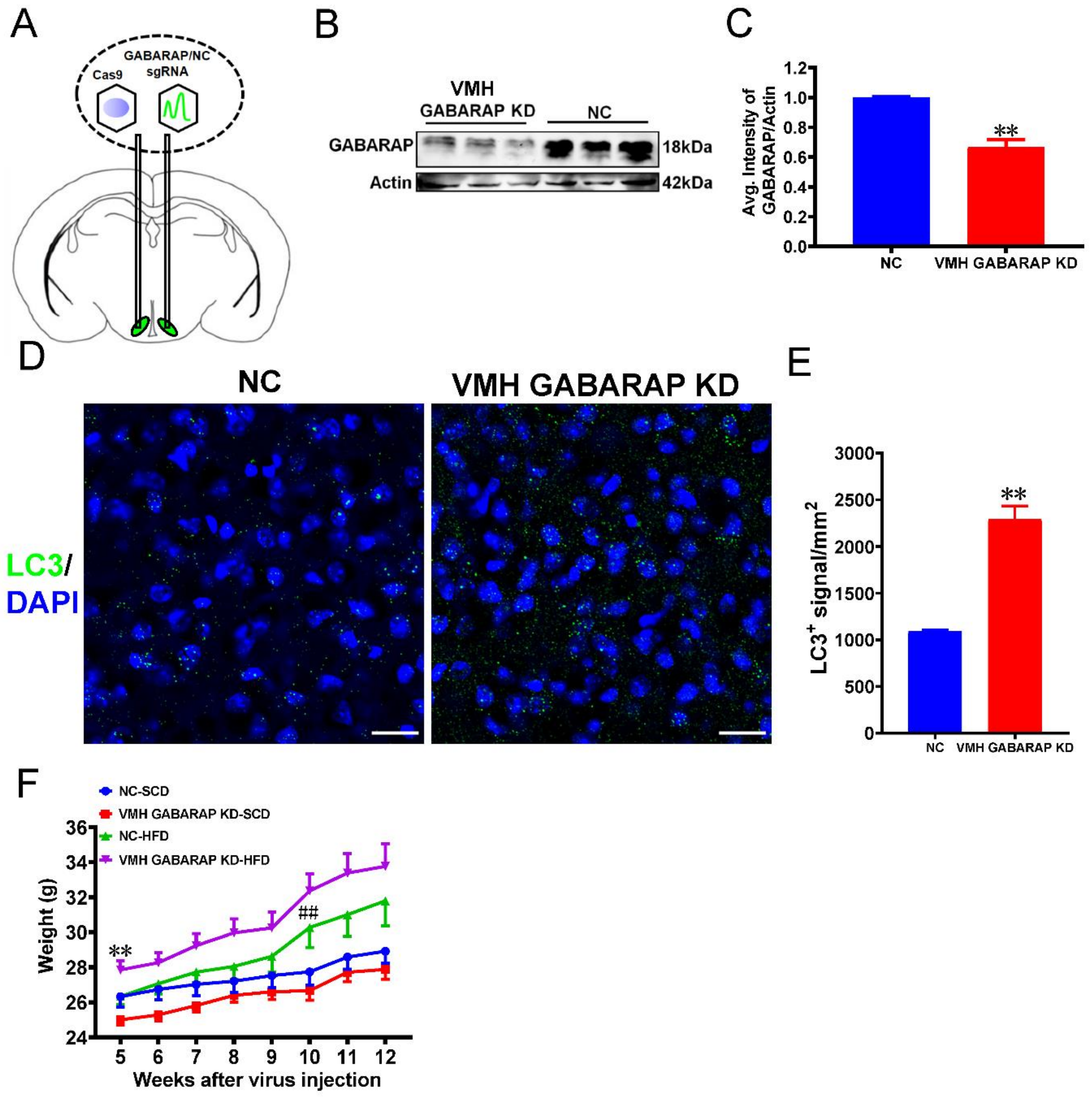

Figure S10. VMH GABARAP KD mice was produced and the autophagy level increased in the VMHs of VMH GABARAP KD mice. A) Schematic representation of the VMH injection of spCas9+GABARAP sgRNAs AAV. B and C) WB (B) and densitometric quantification (C) of GABARAP expression in the hypothalami of VMH GABARAP KD mice and controls (n=3 mice per group). Actin served as the loading control. D) Representative images showing the expression levels of LC3 in the VMHs of VMH GABARAP KD mice and controls. Scale bars, 20 μm. E) Quantification of positive LC3 signals in the VMHs of VMH GABARAP KD mice and controls (n=3 mice per group). F) The growth curves of NC and VMH GABARAP KD

mice starting at 5 weeks after virus injection (n=9 mice per group; **p<0.01 represents the differentiation between NC and VMH GABARAP KD mice under SCD feeding conditions; ##p<0.01 represents the differentiation between NC and VMH GABARAP KD mice under HFD feeding conditions). Data represent the mean ± SEM; **p<0.01; Student's t-test or one-way ANOVA and Bonferroni pairwise comparisons**.**
